# A taxonomic revision of *Pinus* (Pinaceae) in Thailand

**DOI:** 10.7717/peerj.21679

**Published:** 2026-09-03

**Authors:** Chatchai Ngernsaengsaruay, Pichet Chanton, Pichit Lumyai, Nittaya Mianmit, Tharnrat Kaewgrajang, Nisa Leksungnoen, Kritsadapan Palakit, Piyapan Wannamanee

**Affiliations:** 1 Department of Botany, Faculty of Science, Kasetsart University, Chatuchak, Bangkok, Thailand; 2 Biodiversity Center, Kasetsart University (BDCKU), Chatuchak, Bangkok, Thailand; 3 Department of Forest Management, Faculty of Forestry, Kasetsart University, Chatuchak, Bangkok, Thailand; 4 Department of Forest Biology, Faculty of Forestry, Kasetsart University, Chatuchak, Bangkok, Thailand; 5 Forest Industry Organization (FIO), Wat Sommanat, Pom Prap Sattru Phai, Bangkok, Thailand

**Keywords:** Conifers, Bisaccate pollen, Monoecy, Needles, Plicate parenchyma, Tropical pines, Leaf anatomy, Resin ducts, Taxonomy

## Abstract

A taxonomic revision of the native *Pinus* L. (Pinaceae) species in Thailand is presented. Two species, *P. kesiya* Royle ex Gordon and *P. latteri* Mason, are recognized and enumerated with updated morphological descriptions, photographs, and an identification key, together with notes on distribution, habitat and ecology, phenology, conservation status, etymology, vernacular names, uses, and specimens examined. *Pinus kesiya* is neotypified (second-step neotypification), and *P. kasya* Royle ex Parl., a synonym of *P. kesiya*, is lectotypified (second-step lectotypification). *Pinus kesiya* is assessed as Least Concern (LC), whereas *P. latteri* is assessed as Near Threatened (NT). Morphologically, the two species can be distinguished by bark fissuring pattern, thickness, and color; number of needles per fascicle; needle morphology (including rigidity, posture, and shape and diameter in transverse section); length/width ratio of seed cones when closed; apophysis shape and outline; seed scale thickness; relative length of seed wings; and presence or absence of a grass stage. The two species differ ecologically in elevational range and habitat preference, with *P. kesiya* occurring at higher, more humid montane sites and *P. latteri* at lower, drier habitats. *Pinus kesiya* and *P. latteri* differ in needle shape in transverse section, which is triangular in *P. kesiya* and semicircular in *P. latteri*. They also differ in resin duct number and position, with *P. kesiya* having 3–4 external resin ducts near the hypodermis, whereas *P. latteri* has 2–3 medial resin ducts between the endodermis and hypodermis. Pollen morphology is not diagnostic for species delimitation.

## Introduction

Pinaceae Spreng. ex F. Rudolphi is a conifer family within the gymnosperms, classified within the class Pinopsida Burnett, subclass Pinidae Cronquist, Takht. & W. Zimm., and order Pinales Gorozh. (Christenhusz et al., 2011; Yang et al., 2022). It comprises 11 genera and approximately 247 species worldwide (POWO, 2026), and is distributed in North America, extending southward to Nicaragua and the West Indies, as well as across temperate to tropical regions of Eurasia, including Sumatra and the Philippines (Christenhusz, Fay & Chase, 2017).

*Pinus* L. is the largest genus in the family, comprising approximately 127 species worldwide, and is distributed in the Caribbean and the temperate to subtropical regions of the Northern Hemisphere, extending southward to Malesia (POWO, 2026). In China, the genus is represented by 39 species, of which seven are endemic and 16 are introduced (Fu, Li & Mill, 1999). In Vietnam, seven species have been recorded (Phan et al., 2017). In Malesia, the genus is represented by two species, *P. kesiya* Royle ex Gordon and *P. merkusii* Jungh. & de Vriese (*de Laubenfels, 1988*).

*Pinus kesiya* and *P. latteri* are classified within *Pinus* subgenus *Pinus* and section *Pinus*, but belong to different subsections. *Pinus kesiya* is placed in subsect. *Pinus*, whereas *P. latteri* belongs to subsect. *Pinaster* Mayr ex Koehne (Farjon, 2010).

In Thailand, the genus *Pinus* was treated in the Flora of Thailand by Phengklai (1972), who recognized two species, namely *P. kesiya* and *P. merkusii*. The treatment included brief morphological descriptions. Subsequent taxonomic revisions have re-evaluated the identity of the two-needle pine occurring in mainland Southeast Asia. Farjon (2005, 2010) and Businský (2008) recognized that the pine distributed in Myanmar, southern China, Vietnam, Laos, Cambodia, and Thailand should be referred to as *P. latteri* Mason, whereas *P. merkusii sensu* stricto is geographically restricted to northern Sumatra (Indonesia) and the Philippines (Luzon and Mindoro Islands). Under this taxonomic concept, *P. merkusii* does not occur naturally in Thailand, in contrast to earlier treatments of the genus in Thailand. Consequently, Thai records previously referred to *P. merkusii* require reassessment under the current taxonomic framework. This change has important implications for species identification, specimen determination, distributional records, and conservation assessments in Thailand.

The distinction between *P. latteri* and *P. merkusii* has long been controversial due to overlapping morphological characters and conflicting reports on seedling traits, particularly the occurrence of a “grass stage” (Cooling & Gaussen, 1970; Vidaković, 1991). Farjon (2010) noted that most morphological differences between the two taxa are quantitative, primarily involving size-related characters such as needle length and cone dimensions. Nevertheless, the two taxa are currently treated as distinct species with largely allopatric distributions. In the present study, the two-needle pine occurring in Thailand is treated as *P. latteri*, following the taxonomic concepts of Farjon (2005, 2010).

Although the name *P. latteri* has been widely adopted in regional taxonomic treatments, a comprehensive reassessment of Thai *Pinus* has not previously been undertaken. In particular, Thai specimens have not been critically re-examined using an integrated approach that combines field observations, morphology, leaf anatomy, pollen morphology, and updated conservation assessments. As a result, uncertainties remain regarding species delimitation, the interpretation of historical records, and the application of names to Thai material.

In this article, we present a taxonomic revision of the genus *Pinus* in Thailand. We reassess all Thai species based on herbarium specimens, field observations, morphological evidence, leaf anatomical characters, and pollen morphology. We provide updated species descriptions, photographs, illustrations, an identification key, and revised information on distribution, habitat and ecology, phenology, conservation status, etymology, vernacular names, uses, and specimens examined. This revision clarifies species boundaries, updates species distributions and conservation assessments, and facilitates accurate identification of Thai *Pinus*. In addition, a neotype is here designated for *P. kesiya*, and *P. kasya* Royle ex Parl., a synonym of *P. kesiya*, is here lectotypified in a second-step.

## Materials and Methods

### Taxonomic treatment

Field surveys of *Pinus* species were conducted throughout Thailand, encompassing all floristic regions in which native pines are known to occur. Surveys were not undertaken in the south-eastern and peninsular regions because the genus is not naturally distributed there. Voucher specimens collected during this study were identified using regional floras and taxonomic treatments (*e.g*., Phengklai, 1972; de Laubenfels, 1988, Vidaković, 1991; Li, 1997; Fu, Li & Mill, 1999; Nguyen et al., 2004; Farjon, 2005, 2010; Averyanov et al., 2014; Phan et al., 2017). Species identifications were verified through comparison with specimens housed at BKF and with digitized collections available from BM, C, E, G, K, L, M, MO, MW, NY, P, PE, PR, S, UF, and US. Additional distributional records were obtained from the Global Biodiversity Information Facility (GBIF). Herbarium codes follow the Index Herbariorum (Thiers, 2026). Unless otherwise noted, all specimens cited herein were examined directly by the authors. Nomenclatural information, synonymy, and taxonomic interpretations were assembled from relevant literature and verified using the International Plant Names Index (IPNI, 2026), Plants of the World Online (POWO, 2026), and World Flora Online (WFO, 2026). Morphological characteristics, distributions, habitat and ecology, phenology, and uses were described based on both historic and newly collected herbarium specimens, as well as the authors’ observations. Quantitative morphological data were obtained from both herbarium specimens and field observations. Herbarium-based measurements included dimensions (length and width) of needles, pollen cones, seed cones, and seeds. Field-based measurements were additionally used to record bark characteristics and seedling characteristics, including the grass stage. Vernacular names were compiled from the specimens examined and relevant literature (*e.g*., Pooma & Suddee, 2014). Thailand’s floristic regions follow Flora of Thailand Vol. 16(4) (The Forest Herbarium, Department of National Parks, Wildlife and Plant Conservation, 2025). Conservation status assessments were conducted in accordance with the IUCN Red List Categories and Criteria (IUCN Standards and Petitions Committee, 2024), supplemented by GeoCAT analysis (Bachman et al., 2011) and field data. Extent of occurrence (EOO) and area of occupancy (AOO) were calculated in GeoCAT using a 2 × 2 km^2^ grid cell size. All georeferenced occurrence records were compiled from herbarium specimens and field observations, and cultivated or introduced records were excluded prior to analysis. The number of verified occurrence points used for each species is provided in the species treatments. Category assignments (*e.g*., Least Concern, Near Threatened) were based on IUCN criteria thresholds for EOO, AOO, population distribution, and field observations of habitat condition and threats. Permission to collect specimens of *Pinus* was granted by the Department of National Parks, Wildlife and Plant Conservation (Permit No. MNRE 0907.404/25551; 7 December 2023) and the Forest Industry Organization (FIO) (Permit No. MNRE 1408/285; 15 January 2026).

### Anatomical study

Leaf anatomical features of *Pinus* were examined using transverse sections prepared from mature needles. For each species, three individuals were sampled, with three needles collected per individual, representing three different localities. The anatomical sampling was performed separately for each species. For *P. kesiya*, sampled individuals were collected from Mae Hong Son, Chiang Mai, and Loei Provinces, and for *P. latteri*, sampled individuals were collected from Chiang Mai, Loei, and Suphan Buri Provinces. Samples were fixed in FAA and processed using the paraffin method, sectioned at 15–20 μM with a sliding microtome, and stained with fast green. For epidermal studies, the needle epidermis was manually peeled, stained with safranin, and mounted in Permount. Thirty cells per character per individual were measured. Permanent slides were prepared following Johansen (1940). All measurements were calibrated with a stage micrometer. Observations and photomicrographs were made using an Olympus BX53 microscope equipped with an Olympus DP74 digital camera at the Department of Botany, Faculty of Science, Kasetsart University. Anatomical terminology follows Metcalfe & Chalk (1957).

### Palynological study

**Figure 1:**
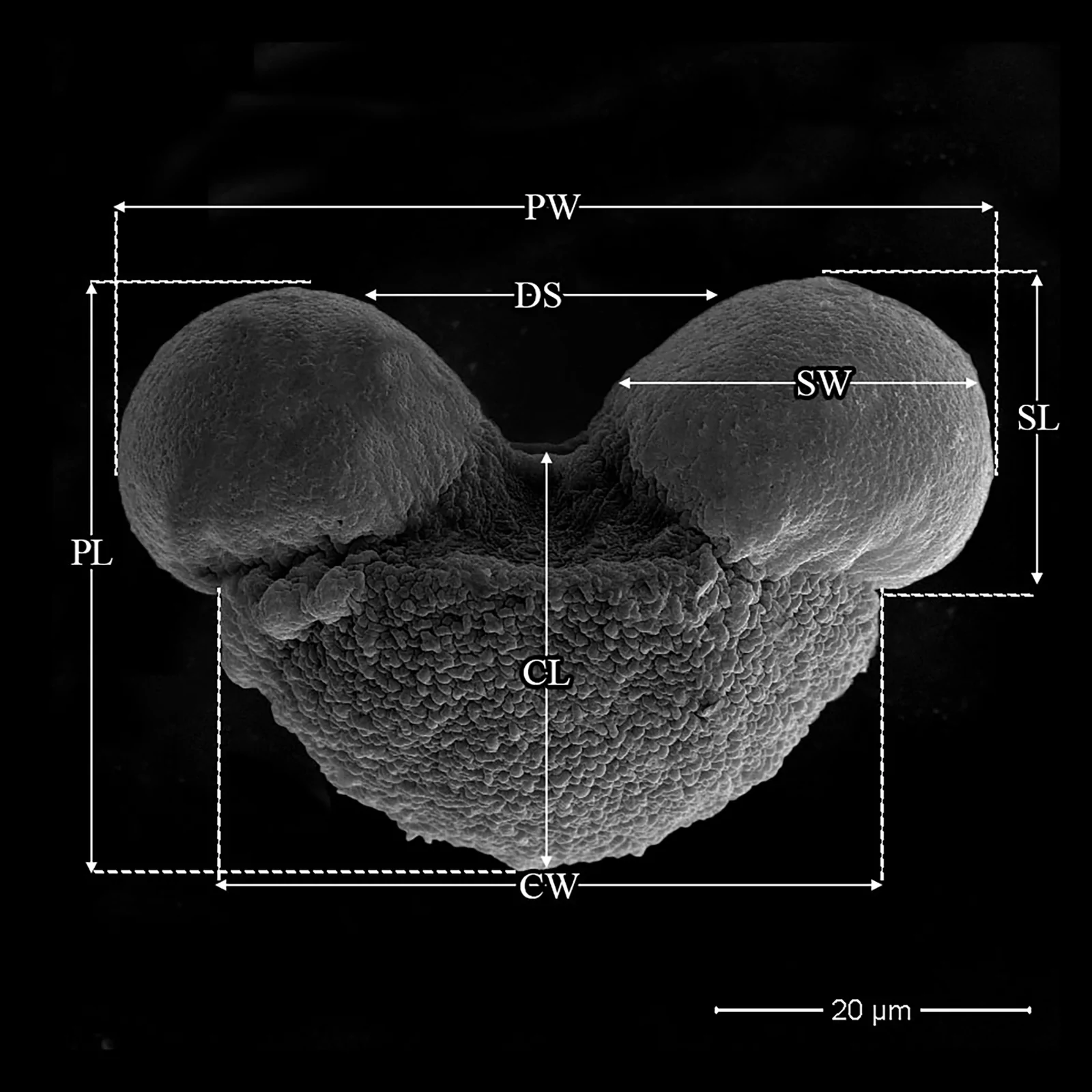
Measurements of bisaccate pollen of the genus *Pinus*. PL, pollen length with sacci; PW, pollen width with sacci; CL, *corpus* length; CW, *corpus* width; SL, saccus length; SW, saccus width; DS, distance between sacci. Image Source Credit: Chatchai Ngernsaengsaruay and Pichet Chanton.

Pollen grains were examined and photographed using an Olympus BX53 light microscope (LM) equipped with an Olympus DP74 digital camera. Observations were conducted without acetolysis to preserve pollen morphology in its natural state, as represented in fresh and herbarium material. For scanning electron microscopy (SEM), pollen grains of both species were examined. Samples were mounted on aluminum stubs using double-sided adhesive tape, sputter-coated with gold, and observed using an FEI Quanta 450 (Hillsboro, OR, USA) operated at 15 kV at the Scientific Equipment Centre, Faculty of Science, Kasetsart University. A total of 30 pollen grains per species were examined under LM and 30 pollen grains per species were also examined under SEM. Specimens representing three different localities for each species were used for both LM and SEM analyses, following the methods of Erdtman (1945, 1952). The following parameters were measured: PL = pollen length with sacci; PW = pollen width with sacci; CL = *corpus* length; CW = *corpus* width; SL = saccus length; SW = saccus width; DS = distance between sacci; ET = exine thickness (Fig. 1). Pollen terminology follows Punt et al. (2007) and Erdtman (1952).

## Results

### Taxonomic treatment

***Pinus* L.**, Sp. Pl. 2: 1000. 1753; Parl. in A. DC, Prodr. 16(2): 378. 1868; Hook. f., Fl. Brit. India 5(15): 651. 1890; Bui, Adansonia T. 2, 2: 332. 1962; Phengklai, Fl. Thailand 2(2): 193. 1972; de Laub., Fl. Males., Ser. 1, Spermat., 10(3): 447. 1988; I. Soerianegara & Lemmens (Eds), PROSEA 50(1): 349. 1993; T. H. Nguyen & J. E. Vidal, Fl. Cambodge, Laos, Vietnam 28: 29. 1995; L. G. Fu *et al*., Fl. China 4: 12. 1999; Farjon, Handb. World's Conifers 1: 608. 2010.

**Type.** *Pinus sylvestris* L.

**Description.** Evergreen monoecious trees; trunk monopodial, straight, cylindrical; resin ducts in bark, wood, leaves, and cones. ***Shoots*** of two types, long and dwarf shoots; cataphylls scale-like, enclosing terminal buds (primordial long shoots or ovuliferous cones), subtending dwarf shoot buds or pollen cones. ***Leaves*** (needles) in fascicles of 2 or 3 on dwarf shoots; fascicles enclosed at base by a persistent sheath, shed as fascicles; acicular, semicircular or triangular in transverse section, margins minutely serrulate. ***Pollen cones*** spirally arranged near proximal end of new long shoots, cylindrical; axis slender; microsporophylls numerous, spirally arranged, subpeltate; pollen sacs 2; pollen bisaccate. ***Ovulate cones*** distinctly smaller than seed cones; axis bearing numerous spirally arranged ovuliferous scales; ovules 2 per scale, adaxial; bracts conspicuous at pollination, not accrescent. ***Seed cones*** subterminal, solitary or in clusters of 2–5, shortly pedunculate, maturing in the second year, persistent, initially erect; mature cones pendulous or spreading, opening at maturity, conical-ovoid, sometimes broadly conical-ovoid, symmetrical or slightly asymmetrical when closed, ovoid, broadly ovoid, or subglobose when open; seed scales attached spirally to the cone axis, persistent, oblong, thin to thick woody; apophysis (exposed portion) thickened and ridged, with a terminal or dorsal umbo (a prominent protuberance), sometimes armed with a spine. ***Seeds*** usually slightly flattened, with a membranous wing. ***Seedlings*** with 7–10 or more cotyledons, with or without a grass stage.

The apophysis is the well-defined apical part of the ovuliferous scale in the seed cone, marking the first stage of growth in *Pinus*; it is rarely differentiated in other genera of the family Pinaceae. The umbo (from Latin, meaning “the boss of a shield”) is a prominence on the apophysis of the ovuliferous scale in pine cones, often armed with a spine (Farjon, 2010).

*Pinus* comprises 127 accepted species worldwide (POWO, 2026), with two native species found in Thailand (*P. kesiya* and *P. latteri*). The genus is widely distributed across North America, parts of North Africa, Europe, and Asia (Farjon, 2010), spanning several continents and climates. These two tropical pines belong to the subgen. *Pinus* and sect. *Pinus*; however, *P. kesiya* is in subsect. *Pinus*, whereas *P. latteri* is in subsect. *Pinaster* (Farjon, 2010).

The generic name *Pinus* is the classical Latin name for pines (Farjon, 2010).

**A key to the species of *Pinus* in Thailand**
1a. Needles in fascicles of 3, lax, drooping, triangular in transverse section, 0.7–1 mm diam.; bark reticulately fissured into irregular, flat, rough plates, 0.3–1 cm thick, grayish brown or reddish brown; seed cones with length/width ratio 1.1–1.9 (closed); apophyses rhombic, irregular in outline; seed scales 2–3.5 mm thick at mid-length; seed wings 1.5–3 times as long as the seed; seedlings without a grass stage **1. *Pinus kesiya***
1b. Needles in fascicles of 2, rigid, straight, semicircular in transverse section, 1.2–1.7 mm diam.; bark deeply fissured longitudinally and transversely, forming irregular, very thick, rough, slightly to distinctly raised plates, 2.5–5.5 cm thick, grayish brown to blackish brown; seed cones with length/width ratio 2.0–2.9 (closed); apophyses rhombic, pentagonal, or hexagonal, irregular in outline; seed scales 4–5.5 mm thick at mid-length; seed wings 3–4.5 times as long as the seed; seedlings with a grass stage **2. *Pinus latteri***

**1. *Pinus kesiya*** Royle ex Gordon, Gard. Mag. & Reg. Rural Domest. Improv. n.s., 6: 8. 1840; Phengklai, Fl. Thailand 2(2): 194. 1972; de Laub., Fl. Males., Ser. 1, Spermat., 10(3): 452. 1988; M. Vidaković, Conifers Morphol. Variat.: 463. 1991; I. Soerianegara & Lemmens (Eds), PROSEA 50(1): 355. 1993; T. H. Nguyen & J. E. Vidal, Fl. Cambodge, Laos, Vietnam 28: 32, pl. 2: 2. 1995; D. Z. Li, Edinburgh J. Bot. 54(3): 345. 1997; L. G. Fu *et al.*, Fl. China 4: 16. 1999; T. H. Nguyen *et al*., Vietnam Conifers: Conservation Status: 75. 2004; Farjon, Pines: Draw. Descr. Genus *Pinus*: 107, fig. 116. 2005 et Farjon, Handb. World's Conifers 1: 691. 2010; Businský, Acta Pruhon. 88: 31, fig. 33A, B, C, 73E. 2008; Aver. *et al*., Nordic J. Bot. 32: 974. 2014; L. K. Phan *et al.*, Pak. J. Bot. 49(5): 2047, fig. 3F. 2017. Type. [India], Khasia, Regio Temp., alt. 2,000–6,000 ft. (“in Khasya alt. 2–6000 ped. in regione temperata et tropica”, protologue), s.d., *J. D. Hooker & T. Thomson s.n*. (neotype: first-step designated by Li (1997), **K** [without barcode]; isolectotype: **E** [without barcode]; neotype: second-step designated here, **K** digital image! [K000289689]; isoneotypes: **C** digital images! [C10020506, C10020507], **E** digital images! [E00078980, E00078981], **K** digital image! [K001081820], **P** digital images! [P00722121, P00722122, P00722123]).

= *Pinus kasya* Royle ex Parl. in A. DC, Prodr. 16(2): 390. 1868, orth. var.—*Pinus khasia* Royle ex Engelm., Trans. Acad. Sci. St. Louis 4: 179. 1880, orth. var.—*Pinus khasya* Royle ex Hook. f. in Hook. f., Fl. Brit. India 5(15): 652. 1890, orth. var. Type. [India], Khasia, Regio Temp., alt. 2,000–6,000 ft. (“in Khasya alt. 2–6000 ped. in regione temperata et tropica”, protologue), s.d., *J. D. Hooker & T. Thomson s.n*. (lectotype: first-step designated by Li (1997), **K** [without barcode]; isolectotype: **E** [without barcode]; lectotype: second-step designated here, **K** digital image! [K000289689]; isolectotypes: **C** digital images! [C10020506, C10020507], **E** digital images! [E00078980, E00078981], **K** digital image! [K001081820], **P** digital images! [P00722121, P00722122, P00722123]).

= *Pinus langbianensis* A. Chev., Rev. Bot. Appl. Agric. Trop. 24: 25, pl. 4: A1, 2, 3. 1944.—*Pinus kesiya* Royle ex Gordon var. *langbianensis* (A. Chev.) Gaussen ex Bui, Adansonia T. 2, 2: 338. 1962.— *Pinus insularis* Endl. var. *langbianensis* (A. Chev.) Silba, Phytologia 68(1): 51. 1990. Type. [Vietnam], Annam, Langbian, Dalat, alt. 1,400 m (“Annam moyen, très commun dans le massif du Langbian”, protologue), 12 Feb 1914, *A. J. B. Chevalier 30024* (lectotype: designated by Bui (1962), **P** digital image! [P00634863]; isolectotype: **S** [S-C-2816], no image available).

= *Pinus insularis* Endl. var. *khasyana* (Griff.) Silba, Phytologia 68(1): 51. 1990.— *Pinus khasyana* Griff., Itin. Pl. Khasyah Mts.: 58. 1848 (as *Pinus*); Not. Pl. Asiat. 4: 18. 1854 (as *P. khasyanus*).

**Figure 2:**
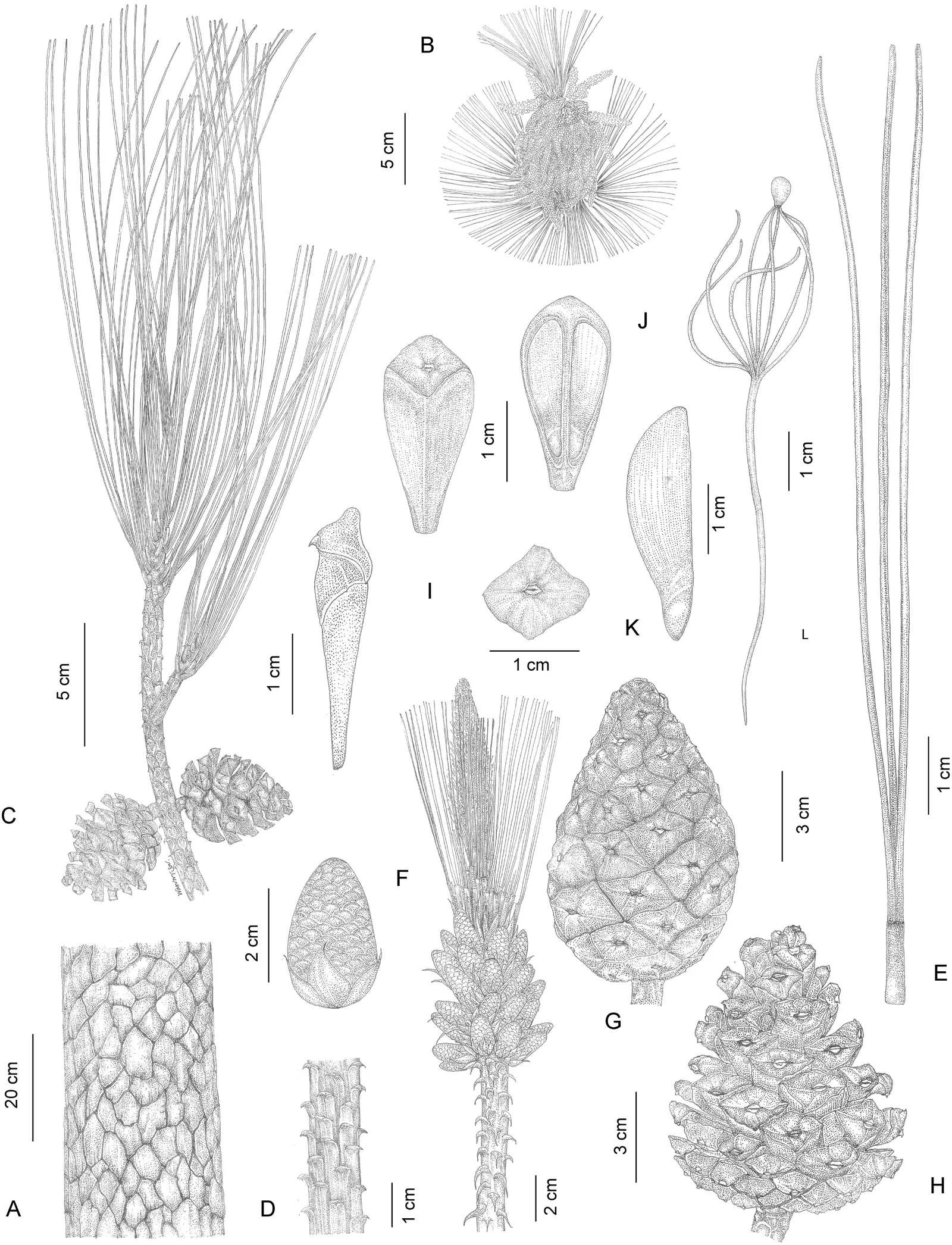
*Pinus kesiya*. (A) Stem and outer bark. (B and F) Branchlets, leaves, and pollen cones. (C) Branchlets, leaves, and open seed cones. (D) Branchlet showing scars of fallen needle fascicles. (E) Needle fascicle. (G) Closed seed cone. (H) Open seed cone. (I) Seed scales. (J) Seed scale and winged seeds. (K) Winged seed. (L) Seedling with several cotyledons. Image Source Credit: Wanwisa Bhuchaisri.

**Figure 3:**
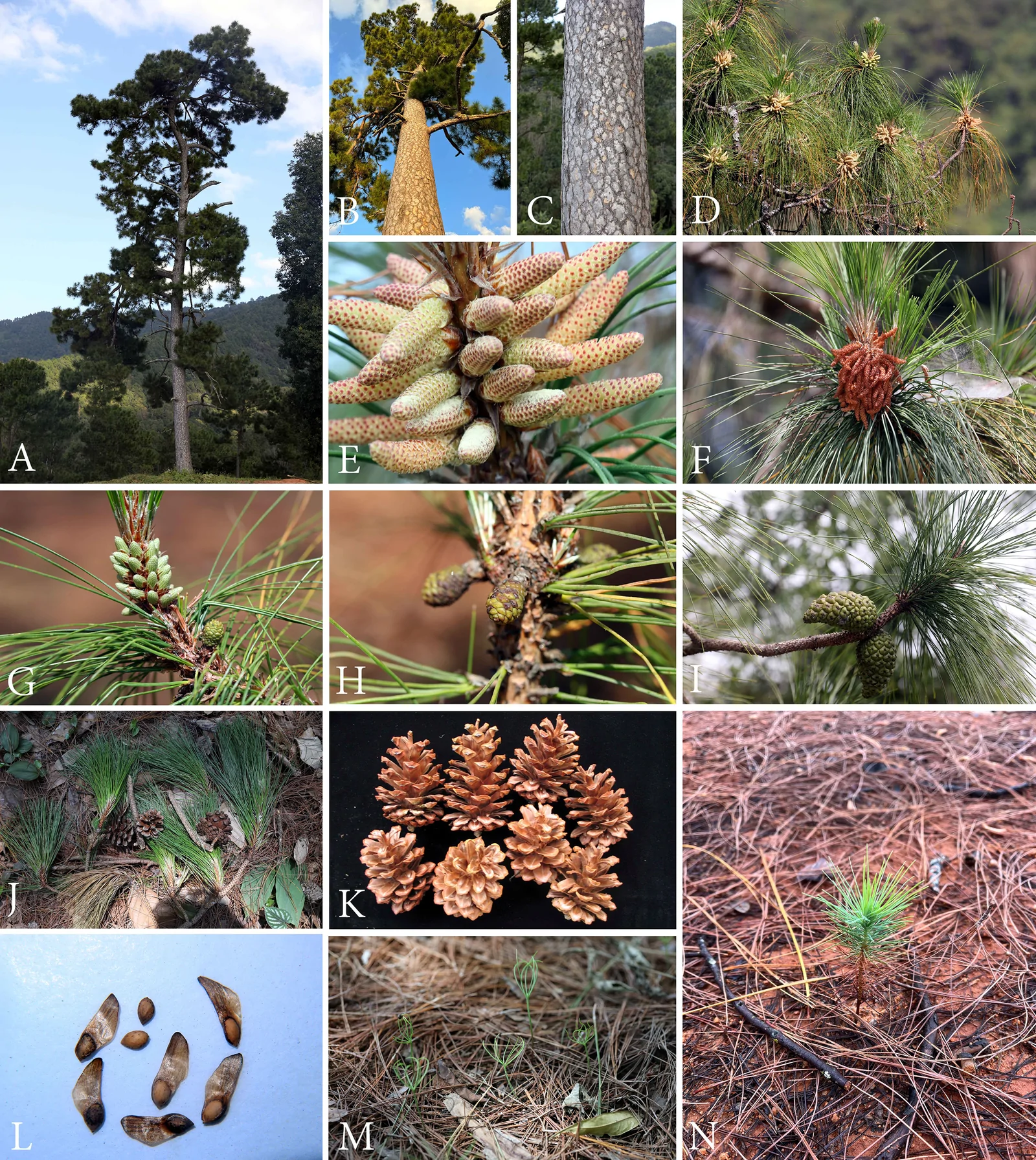
*Pinus kesiya*. (A and B) Habit. (C) Stem and outer bark. (D–F) Branchlets, leaves, and pollen cones. (G) Branchlets, leaves, pollen cones, and ovulate cone. (H) Branchlets, leaves, and ovulate cones. (I and J) Branchlets, leaves, and seed cones (closed and open). (K) Open seed cones. (L) Winged seeds. (M) Seedlings with several cotyledons. (N) Seedling without a grass stage. Image Source Credit: Chatchai Ngernsaengsaruay (A–K and M, N); Pichet Chanton (L).

**Description.** Trees 10–35(–45) m tall; trunk usually straight, cylindrical, 50–330 cm GBH. ***Bark*** reticulately fissured into irregular, flat, rough plates, 0.3–1 cm thick, grayish brown or reddish brown. ***Branches*** spreading to ascending, usually crooked, crown irregular, broadly domed, umbrella-shaped, or flat-topped. ***Foliage branchlets*** slender, rough from leaf fascicle scars. ***Terminal buds*** small, conical; cataphylls reddish brown. ***Leaves*** in fascicles of 3, basal sheath persistent, reddish brown to brown, 7.5–15 × 1–1.5 mm; needles (10–)13–25 cm × 0.7–1 mm, green, glabrous, lax, drooping, triangular in transverse section, apex acuminate, margins minutely serrulate. ***Pollen cones*** clustered, spirally arranged at the base of new shoots, cylindrical, 1.6–2.5 × 0.5–0.9 cm, pale green when young, yellow to brownish yellow at maturity, brown when dry. ***Ovulate cones*** ovoid, broadly ovoid, subglobose, or obovoid, 1.3–2 × 1.2–1.8 cm, green. ***Seed cones*** solitary or in clusters of 2–3, shortly pedunculate, spreading or reflexed, persistent; closed cones conical-ovoid, sometimes broadly conical-ovoid, symmetrical or slightly asymmetrical, 5–8 × 3–5 cm (length/width ratio 1.1–1.9); open cones broadly ovoid or subglobose, 5–8 × 5–6.5 cm (length/width ratio 1.0–1.4); seed scales green when immature, lustrous, reddish brown at maturity, thin woody, more or less recurved when spreading, oblong, 2–3.5 × 1–1.5 cm, 2–3.5 mm thick at mid-length; apophyses prominently raised, more or less pyramidal, rhombic, irregular in outline, distinctly transversely or radially ridged, central part shallowly concave; umbo small, usually sunken, elliptic in outline, sometimes slightly protruding, armed with a tiny recurved deciduous spine. ***Seeds*** ellipsoid, 6–8.5 × 4–5 mm, reddish brown to dark brown; wing articulated to seed, 1.1–2.5 × 0.6–0.8 cm, membranous, persistent, 1.5–3 times as long as the seed. ***Seedlings*** with 7–8 or more cotyledons, without a grass stage (Figs. 2 and 3).

**Figure 4:**
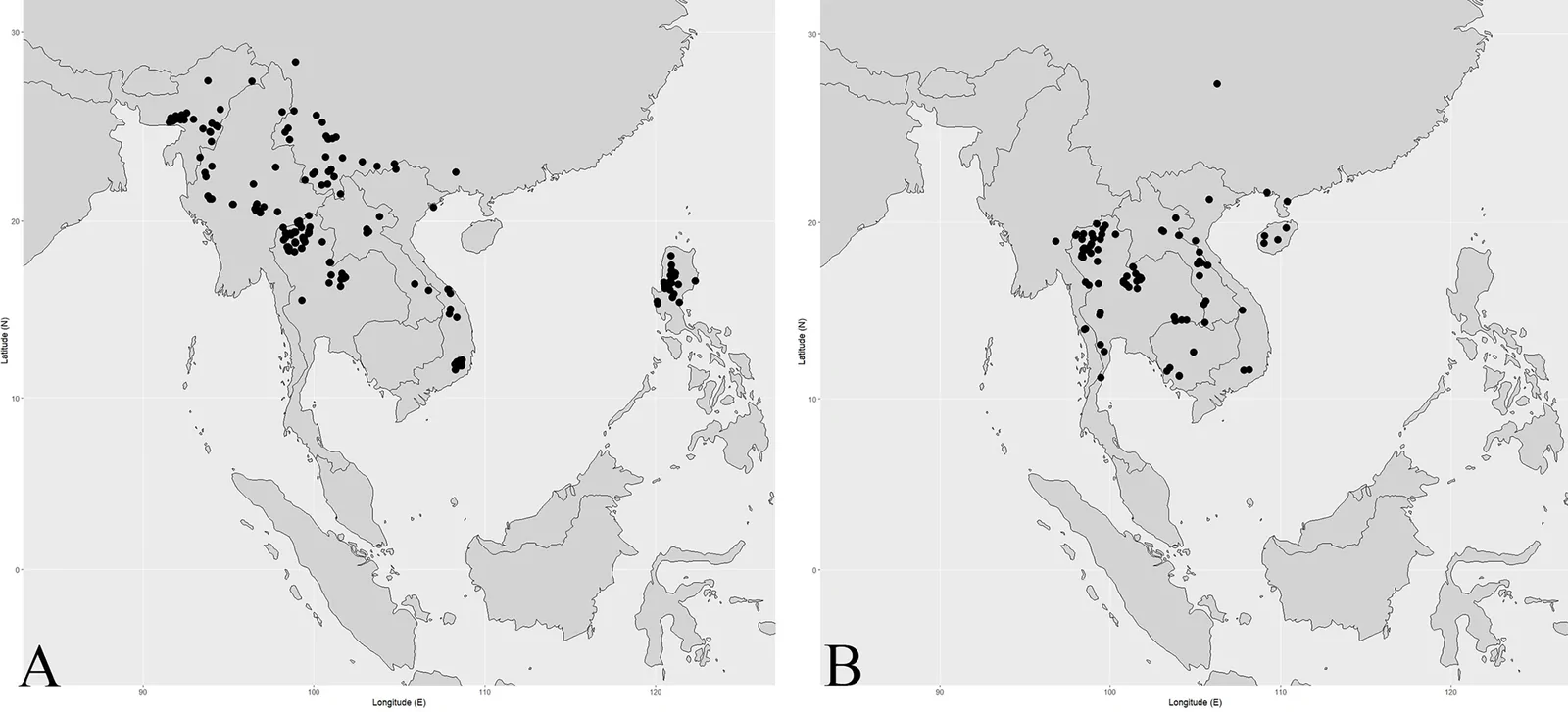
Distribution of two Thai *Pinus* species. (A) *Pinus kesiya* is distributed in India (Assam), Myanmar, China (Yunnan; Xizang), Vietnam, Laos, Cambodia, Thailand, and the Philippines (Luzon). In Thailand, this species is distributed in the northern, north-eastern, eastern, and south-western regions. (B) *Pinus latteri* is distributed in Myanmar, China (Guangdong; Guangxi), Vietnam, Laos, Cambodia, and Thailand. In Thailand, this species is distributed in the northern, north-eastern, eastern, south-western, and central regions. Map Source Credit: Pichet Chanton and Chatchai Ngernsaengsaruay.

**Distribution.** India (Assam), Myanmar, China (Yunnan; Tibet: south-eastern Xizang), Vietnam, Laos, Cambodia, Thailand, and Philippines (Luzon) (Fig. 4A).

**Distribution in Thailand. Northern:** Mae Hong Son (Pai, Pang Ma Pha, Huai Nam Dang), Chiang Mai (Doi Suthep-Pui, Doi Inthanon, Doi Chiang Dao, Doi Pha Hom Pok, Doi Ang Khang, Doi Wiang Pha, Huai Nam Dang, Kalayaniwattana, Ban Wat Chan), Chiang Rai (Doi Tung, Khun Chae, Doi Langka, Doi Pha Ngom, Doi Mot), Phayao (Mae Chai: Doi Luang, Tham Prakai Phet), Nan (Ban Luang), Lamphun (Mae Tha: Doi Khun Tan; Ban Hong: Doi Chang), Lampang (Chae Son, Wang Nuea: Doi Luang), Uttaradit (Phu Soi Dao), Tak (Taksin Maharat), Phitsanulok (Phu Hin Rong Kla); **North-Eastern:** Phetchabun (Nam Nao, Thung Salaeng Luang), Loei (Phu Kradueng, Phu Ruea, Phu Luang); **Eastern:** Chaiyaphum (Phu Khiao); **South-Western:** Uthai Thani (Huai Kha Khaeng) (Fig. 4A).

**Figure 5:**
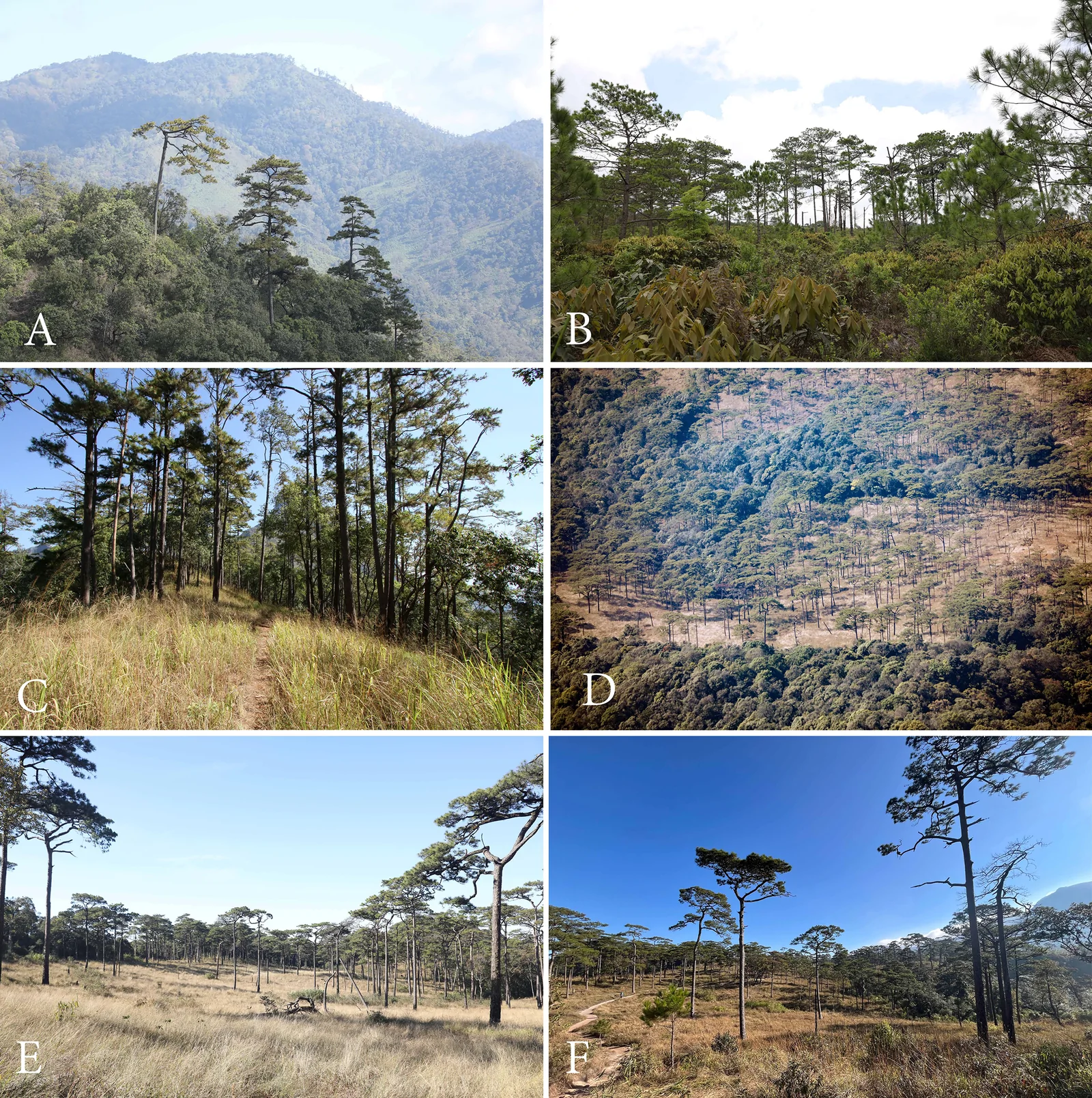
Habitat and ecology of *Pinus kesiya* in Thailand. (A) Lower montane pine-oak forest at Doi Langka, Chiang Rai Province, at an elevation of ca. 1,700 m. a.m.s.l. (B) Lower montane pine-oak forest at Phu Luang, Loei Province, at an elevation of ca. 1,490 m. a.m.s.l. (C) Lower montane pine-oak forest at Doi Chiang Dao, Chiang Mai Province, at an elevation of ca. 1,375 m. a.m.s.l. (D–F) Lower montane pine-oak forest (pine-oak savanna-like structure) at Phu Soi Dao, Uttaradit Province, at an elevation of ca. 1,600 m. a.m.s.l. Image Source Credit: Chatchai Ngernsaengsaruay.

**Habitat and ecology**. *Pinus kesiya* occurs in lower montane pine-oak forests and pine-deciduous dipterocarp forests at elevations of 800–1,800 m a.m.s.l. In Thailand, the lowest elevation record of *P. kesiya* is from pine-deciduous dipterocarp forest along the Nong Mae Na–Thung Nang Phaya route, Thung Salaeng Luang National Park, Khao Kho District, Phetchabun Province, at approximately 800 m a.m.s.l. Its highest elevation record in lower montane pine-oak forest is from Doi Mot in Khun Chae National Park, Chiang Rai Province, at about 1,800 m a.m.s.l. Lower montane pine-oak forests in some localities may exhibit a pine (*P. kesiya*)-oak savanna-like structure, such as at Phu Soi Dao (ca. 1,600 m a.m.s.l.) and Phu Kradueng (ca. 1,200 m a.m.s.l.) (Fig. 5).

**Phenology.** Pollen cones (September–November), mostly December–February; ovulate cones (November–) February–April; pollination during the dry season; fertilization following pollination; seed cones present throughout the year, requiring 2 years to mature.

**Conservation status.** *Pinus kesiya* is widely distributed from India (Assam) to China (Yunnan), Indo-China, and the Philippines. It has been recorded from numerous localities and has a large extent of occurrence (EOO) of 3,293,865.98 km^2^ and an area of occupancy (AOO) of 712 km^2^. In Thailand, it occurs naturally in many localities across four floristic regions, with an EOO of 162,395.85 km^2^ and an AOO of 308 km^2^. Based on its wide distribution, numerous localities, and large EOO, the species is not currently considered to face significant threats. We therefore assess its conservation status as Least Concern (LC), following Nguyen et al. (2004) and Farjon (2010, 2013).

**Etymology.** The specific epithet of *P. kesiya* is derived from the Khasi Hills of northeastern India, from which the species was first reported by J. F. Royle. The spelling of the specific epithet has long been the subject of unnecessary controversy. However, Royle’s European spelling of the toponym, as used at the time, is no less valid than any alternative and should therefore be followed in the botanical nomenclature of this species (Farjon, 2010).

**Vernacular names.** Kia plueak daeng (เกี๊ยะเปลือกแดง) (Northern); Kia plueak bang (เกี๊ยะเปลือกบาง) (Chiang Mai); Chuang (จ๋วง) (Northern, North-eastern); Chiang-bang (เชี้ยงบั้ง) (Karen-Mae Hong Son); Paek (แปก) (Phetchabun, Shan-Mae Hong Son); Son khao (สนเขา), **Son sam bai** (**สนสามใบ**) (Central); Dingsa, Far, Saral (India) (Farjon, 2010); Tinyu (Myanmar) (Farjon, 2010); Ka xi song (Chinese) (Fu, Li & Mill, 1999); Thông ba lá (Vietnamese) (Nguyen et al., 2004); Benguet pine (Farjon, 2005, 2010), Khasi pine (Silba, 1990; Farjon, 2005, 2010), Khasia pine (Li, 1997; Farjon, 2005, 2010), Khasya pine (Nguyen et al., 2004), Langbian pine (Silba, 1990), Luzon pine (Farjon, 2005, 2010), Three-needled pine (English).

**Uses.** *Pinus kesiya* is a multipurpose tree species with significant economic, ecological, and medicinal value. Its wood is used in residential construction, furniture manufacturing, and the production of household utensils. It is also utilized as firewood and for charcoal production, and serves as an ignition fuel. The wood possesses suitable properties for pulp and paper production. Pine resin is used in the manufacture of varnish and as rosin for the bows of stringed musical instruments. In addition, the wood, leaves, and resin are traditionally used in local herbal medicine. This species is planted for forest restoration and watershed rehabilitation and is also cultivated as an ornamental tree.

**Typifications**. *Pinus kasya* Royle ex Parl. was published by Parlatore (1868), with the protologue citing the type locality as “In Nepalia? in Khasya alt. 2–6000 ped. in regione temperata et tropica (*Griffith! n. 4995*, Hook. fil. et Th.!)”. Later, the names *P. khasia* Royle ex Engelm. (Engelmann, 1880) and *P. khasya* Royle ex Hook. f. (Hooker, 1888) were published. These three names represent orthographical variants of the same taxon and are based on the same type material.

The name *P. kasya* was lectotypified in a first step by Li (1997) on the specimen *J. D. Hooker & T. Thomson s.n*., designating the specimen at K [without barcode] as the lectotype and that at E [without barcode] as the isolectotype. However, upon examination, it was found that multiple specimens of this gathering are preserved at C [C10020506, C10020507], E [E00078980, E00078981], K [K001081820, K000289689], and P [P00722121, P00722122, P00722123]. Therefore, the specimen at K [K000289689] is here designated as the second-step lectotype, and the remaining specimens are treated as isolectotypes, following Art. 9.17 of the ICN (Turland et al., 2018).

*Pinus kesiya* Royle ex Gordon was published in 1840 (Gordon, 1840), and it is most likely that no original herbarium material was preserved at that time, as the species was raised from seeds presented to the Society by Dr. Royle, F. H. S. (Li, 1997). Li (1997) designated a neotype based on the gathering *J. D. Hooker & T. Thomson* s.n., the same specimen designated as the lectotype of *P. kasya*. Subsequent examination revealed that multiple specimens of this gathering are preserved at C [C10020506, C10020507], E [E00078980, E00078981], K [K001081820, K000289689], and P [P00722121, P00722122, P00722123]. Therefore, the specimen at K [K000289689] is here designated as the second-step neotype, and the remaining specimens are treated as isoneotypes, following Art. 9.17 of the ICN (Turland et al., 2018).

*Pinus langbianensis* was described by Chevalier (1944), who cited the type locality in the protologue as: “*Annam moyen*: très commun dans le massif du Langbian (*Chevalier 30024*, type), fév. 1914”. However, Chevalier did not specify the category of the type nor the herbarium in which the type specimen was deposited; consequently, no holotype was effectively designated at the time of publication. Subsequently, Bui (1962) referred to the same gathering, *Chevalier 30024*, as the type and indicated that the specimen is housed at P, but did not specify the type category or a specimen barcode; in the absence of a holotype designation in the protologue, this is interpreted as an implicit lectotypification under Art. 9.10 of the ICN and should therefore be followed (Turland et al., 2018). The later treatment by Li (1997), who referred to the specimen as a “holotype”, is not consistent with the ICN, since a holotype can only be established when explicitly designated in the protologue. Our study confirms the existence of the lectotype at P (digital image available: P [P00634863]) and an isolectotype at S (S [S-C-2816], no image available).

**Notes.** *Pinus kesiya* is morphologically similar to *P. yunnanensis* but differs in several diagnostic characters. *Pinus kesiya* has needles in fascicles of three only; the needles are lax and drooping, usually less than 1 mm diam. Its apophyses are more or less pyramidal and distinctly transversely or radially ridged. In contrast, *P. yunnanensis* bears needles in fascicles of three, occasionally two; the needles are stiff and straight, 1–1.2 mm diam. The apophyses are swollen, generally not pyramidal, and not or only slightly transversely ridged. The morphological characters of *P. yunnanensis* are based on Farjon (2010) and Fu, Li & Mill (1999).

Farjon (2010), Fu, Li & Mill (1999), and Li (1997) further noted that *P. kesiya* has bi- or multi-nodal new foliage shoots, producing more than one whorl of branches or buds in a single growing season, and marginal resin ducts. In contrast, *P. yunnanensis* has uni-nodal new foliage shoots and resin ducts that are both marginal and median.

According to Farjon (2010), *P. kesiya* occupies drier sites in northeastern India, Myanmar and Thailand, at elevations generally from 800 to 1,500 m a.m.s.l., occasionally reaching 2,000 m a.m.s.l. Further east, in Laos, Vietnam and on the island of Luzon in the Philippines, its elevation range is broader, extending from 2,700 to 3,000 m a.m.s.l., where it occurs in a much wetter climate.

**Additional specimens examined. THAILAND. Northern: Mae Hong Son** [Pang Ma Pha, seed cone, 2 May 2009, *M. Hilscher 38* (L [L.4320773]); Huai Nam Dang National Park, ♂ cone, 5 Feb 2013, *M. Norsaengsri 10144* (BKF); Huai Wai Forest Ranger Unit (Temporary), Mae Lao-Mae Sae Wildlife Sanctuary, Pai District, lower montane pine-oak forest, ca. 1,460 m a.m.s.l., 7 Nov 2022, *C. Ngernsaengsaruay* personal observation with photos; Pai District, lower montane pine-oak forest, ca. 1,160 m a.m.s.l., 27 May 2023, *C. Ngernsaengsaruay* personal observation with photos]; **Chiang Mai** [Doi Suthep, seed cone, 1 Jan 1905, *C. C. Hosseus 318* (K [K000554160], L [L.1183133], M [M-0168819, M-0168820]); ibid., seed cone, 30 Jan 1910, *A. F. G. Kerr 962* (K [K000554162]); ibid., seed cone, 1 Nov 1920, *J. F. Rock 163* (US [02065554]), *J. F. Rock 247* (US [02065566]); ibid., ♂ cone, 28 Sep 1949, *Ploenchit 48* (BKF); ibid., seedling, 4 May 1958, *T. Sørensen et al. 3235* (BKF); ibid., seed cone, 29 Dec 1988, *J. F. Maxwell 88-1416* (BKF, L [L.4178500, L.4178501]); ibid., ♂ cone, 2 Feb 1989, *J. F. Maxwell 89-141* (BKF, L [L.1183161], MO [976290], UF [268802]); Khun Chang Khian, seed cone, 13 Mar 1930, *Phraya Phananuchon 4* (BKF); Doi Pui, seed cone, 27 Nov 1984, *H. Koyama T-49525* (BKF); ibid., seed cone, May 1987, *C. Phengklai et al. 6330* (BKF); ibid., 8 Aug 1988, *M. N. Tamura T-60755* (BKF); Doi Suthep-Pui National Park, seed cone, 14 Jul 1988, *H. Koyama T-61910* (BKF); ibid., ♂ cone & seed cone, 10 Feb 1999, *C. Kuarak 13* (L [L.4169890]); Doi Pui, lower montane pine-oak forest, ca. 1,400 m a.m.s.l., 24 Apr 2012, *C. Ngernsaengsaruay* personal observation with photos; Doi Inthanon National Park, 16 Jan 1905, *C. C. Hosseus 347b* (M [M-0168821]); ibid., 28 Sep 1949, *P. Suvarnakoses 48* (BR [BR0000026050065V]); Mae Chaem, 25 Jan 1964, *B. Hansen et al. 11110* (L [L.1183118]); Mae Pan Waterfall, seed cone, 29 Jul 1988, *N. Fukuoka T-62312* (BKF); Chom Thong District, ♂ cone, 15 Jan 1993, *J. F. Maxwell 93-40* (BKF, L [L.4178640, L.4178641]); ibid., ♂ cone, 3 Feb 1998, *F. Konta & C. Phengklai 3911* (BKF); ibid., 6 May 2010, *P. Georgiadis 315* (L [L.2058679]); Doi Inthanon, ♂ cone, 17 Dec 1993, *C. Yonebayashi 93039* (BKF); ibid., ♂ cone, 3 Feb 1998, *F. Konta & S. Khao-iem* 10822 (BKF); Doi Pha Tang, Doi Inthanon National Park, lower montane pine-oak forest, ca. 1,040 m a.m.s.l., seed cone, 25 May 2023, *C. Ngernsaengsaruay* personal observation with photos; Doi Inthanon National Park, lower montane pine-oak forest, ca. 1,500 m a.m.s.l., 25 May 2023, *C. Ngernsaengsaruay* personal observation with photos; Doi Chiang Dao, ♂ cone, 24 Dec 1931, *Put 4529* (K [K000554199]); ibid., 30 Jul 1968, *K. Larsen et al. 2852* (BKF); ibid., seed cone, 15 Nov 1988, *W. Nanakorn & H. T. Beck 880171* (MO [103464246], NY [00039940]); ibid., 23 Oct 1992, *R. Pooma 696* (BKF); ibid., 1 Mar 1995, *J. F. Maxwell 95-165* (BKF, L [L.4178639]); San Pa Kia, 23 Jan 1981, *Forestry student s.n*. (BKF [72298]); Doi Chiang Dao, lower montane pine-oak forest, ca. 1,375 m a.m.s.l., 3 Dec 2002, *C. Ngernsaengsaruay* personal observation with photos; Doi Ang Khang, seed cone, 26 May 1998, *T. Wongprasert s.n*. (BKF [123797]); Ban Huai Khiat Haeng, Kalayaniwattana District, 8 Mar 2018, *W. Pongamornkul 6594* (BKF)]; **Chiang Rai** [Doi Tung, ♂ cone, 14 Feb 1983, *H. Koyama et al. T-33532* (BKF); Khun Chae National Park, seed cone, 12 Nov 1997, *U. Wongchai WU039* (BKF); Khun Chae National Park, Wiang Pa Pao District, pine-deciduous dipterocarp forest, 920–930 m a.m.s.l., seed cone, 28 May 2023, *C. Ngernsaengsaruay* personal observation with photos; Doi Langka nature trail (Doi Pha Ngom, Doi Langka Noi), Khun Chae National Park, Wiang Pa Pao District, lower montane pine-oak forest, 1,540–1,770 m a.m.s.l., 26–28 Dec 2025, *C. Ngernsaengsaruay* personal observation with photos]; **Phayao ** [Tham Prakai Phet, Mae Chai District, seed cone, 26 Jul 2016, *N. Muangyen 254* (BKF); Doi Luang National Park, ♂ cone, 9 Feb 2016, *N. Muangyen 663* (BKF)]; **Lamphun** [Doi Khun Tan National Park, Mae Tha District, seed cone, 5 Sep 1967, *M. Tagawa et al. T-9260* (L [L.1183124]); ibid., ♂ cone, 31 Jan 1994, *J. F. Maxwell 94-158* (BKF, L [L.4178589]); Doi Chang, Ban Pa Pae, Pa Phlu Subdistrict, Ban Hong District, pine-deciduous dipterocarp forest, ca. 1,100 m a.m.s.l., 22 Apr 2012, *C. Ngernsaengsaruay* personal observation with photos]; **Lampang** [Chae Son National Park, ♂ cone & seed cone, 31 Jan 1997, *F. Maxwell 97-97* (BKF, L [L.4169572], MO [1775345]); Doi Luang National Park, Wang Nuea District, seed cone, 10 Dec 2002, *I. Schanzer N02-115* (BKF)]; **Uttaradit** [Phu Soi Dao National Park, seed cone, 30 Nov 20xx, *N. Pan-in PSD013* (BKF); ibid., ♂ cone, 22 Feb 2018, *N. Pan-in PSD026* (BKF); Phu Soi Dao National Park, Nam Pat District, lower montane pine-oak forest, ca. 1,600 m a.m.s.l., 4 Jan 2025, *C. Ngernsaengsaruay personal observation with photos*]; **Phitsanulok** [Air Raid Shelter, Phu Hin Rong Kla National Park, Nakhon Thai District, lower montane pine-oak forest, ca. 1,200 m a.m.s.l., ♂ cone & seed cone 2 Feb 2007, *C. Ngernsaengsaruay* personal observation with photos]; **North-Eastern: Phetchabun** [Nam Nao, ♀ cone, 1 Apr 1953, *Sanong 36* (BKF); Locality unspecified, ♀ cone, 10 Apr 1976, *B. Sangkhachand & C. Phengklai 3118*, (BKF, L [L.1183134], K [K000554158]); Nong Mae Na–Thung Nang Phaya route, Thung Salaeng Luang National Park, Khao Ko District, pine-deciduous dipterocarp forest, 750–900 m a.m.s.l., 29 Jan 2007, *C. Ngernsaengsaruay* personal observation with photos]; **Loei** [Phu Kradueng National Park, ♀ cone, 12 Mar 1924, *A. F. G. Kerr 8740* (K [K000554201]); ibid., 12 Jan 1960, *L. B. Abbe et al. 9464* (BKF, NY [4278102]); ibid., seed cone, 28 Nov 1965, *M. Tagawa et al. T-507* (BKF, L [L.1183125]); ibid., seed cone, 30 Nov 1965, *M. Tagawa et al. T-959* (BKF, L [L.1183126]); ibid., 19 Dec 1982, *H. Koyama et al. T-31369* (BKF); ibid., seed cone, 2 Nov 1984, *G. Murata et al. T-42876* (BKF); ibid., seed cone, 4 Sep 1988, *H. Koyama T-61543* (L [L.4168389]); ibid., seed cone, 26 Aug 1954, *T. Smitinand 1881* (BKF); Phu Kradueng National Park, lower montane pine-oak forest, ca. 1,200 m a.m.s.l., seed cone, 13 Nov 2021, *C. Ngernsaengsaruay* personal observation with photos; Phu Ruea National Park, lower montane pine-oak forest, ca. 1,360 m a.m.s.l., 12 Jun 2023, *C. Ngernsaengsaruay* personal observation with photos; Phu Luang, ♂ cone & seed cone, 1 Oct 1966, *E. Hennipman 3601* (K [K000554159], L [L.1183123]); ibid., seed cone, 8 Jun 2015, *S. Tagane T4491* (BKF); Phu Luang Wildlife Sanctuary, lower montane pine-oak forest, 1,470–1,490 m a.m.s.l., seed cone, 10 Jun 2023, *C. Ngernsaengsaruay* personal observation with photos].

**2. *Pinus latteri*** Mason, J. Asiat. Soc. Bengal 18(1): 74. 1849; Phengklai, Fl. Thailand 2(2): 193. 1972 (as *P. merkusii*); Hook. f., Fl. Brit. India 5(15): 652. 1890 (as *P. merkusii*); M. Vidaković, Conifers Morphol. Variat.: 476. 1991 (as *P. merkusii*); T. H. Nguyen & J. E. Vidal, Fl. Cambodge, Laos, Vietnam 28: 35. 1995 (as *P. merkusii*); L. G. Fu *et al.*, Fl. China 4: 17. 1999; T. H. Nguyen *et al.*, Vietnam Conifers: Conservation Status: 81. 2004; Farjon, Pines: Draw. Descr. Genus *Pinus*: 115, fig. 114. 2005 et Farjon, Handb. World's Conifers 1: 698, fig. 227. 2010; Businský, Acta Pruhon. 88: 9, fig. 28E, F. 2008, Phyton (Horn) 54(1): 7, fig. 4. 2014 et Phyton (Horn) 56(2): 147. 2016; Aver. et al., Nordic J. Bot. 32: 975. 2014; L. K. Phan *et al.*, Pak. J. Bot. 49(5): 2048, fig. 4A, B. 2017.— *Pinus merkusii* Jungh. & de Vriese var. *latteri* (Mason) Silba, Phytologia 68(1): 53. 1990.*— Pinus merkusii* Jungh. & de Vriese subsp. *latteri* (Mason) D. Z. Li, Edinburgh J. Bot. 54(3): 346. 1997. Type. Thailand, Chiang Mai Province, Chiang Dao District, Doi Chiang Dao, 13 May 2009, *R. Businský 67116* (neotype: designated by Businský (2014), **PR**; isoneotypes: **BM**, **G**, **L**, **MO**, **P**).

= *Pinus finlaysoniana* Wall. [Numer. List: 207. Wallich Cat. 6062. 1831–1832] ex Blume, Rumphia 3: 210. 1847, nom. illeg.

= *Pinus ikedae* Yamam., Contrib. Fl. Kainan. 1: 20, t. 1. 1943 (as *P. ikedai*). Type. [China], Hainan, Kun Yam Ngan, Lin Fa Shaan, 9 Aug 1927, *W. T. Tsang 418* [Lingnan Univ., Canton No. 15917], det. as *P. merkusii* by E. D. Merrill (lectotype: designated by Businský (2014), **PE**).

= *Pinus tonkinensis* A. Chev., Rev. Bot. Appl. Agric. Trop. 24: 29. pl. 4: D7, 8. 1944.— *Pinus merkusii* Jungh. & de Vriese var. *tonkinensis* (A. Chev.) Gaussen ex Bui, Adansonia T. 2, 2: 336. 1962.— *Pinus latteri* Mason subsp. *tonkinensis* (A. Chev.) Silba, J. Int. Conifer Preserv. Soc. 16(1): 25. 2009. Type. [Vietnam], Annam, Nghệ An Province (Vinh), Hoan-Mai Forest Reserv (“Nord Annam, Province de Nghé-An (Vinh), réserve forestière de Hoan-Mai”, protologue), s.d., *J. F. Fleury s.n*. (in Herb. Chevalier 30202) (lectotype: designated by Businský (2014), **P** digital image! [P00731197]).

= *Pinus merkusiana* Cooling & Gaussen, Trav. Lab. Forest. Toulouse T. 1, 8(7): 5. 1970, without indication of the type, nom. inval. (Art. 37.1).

**Figure 6:**
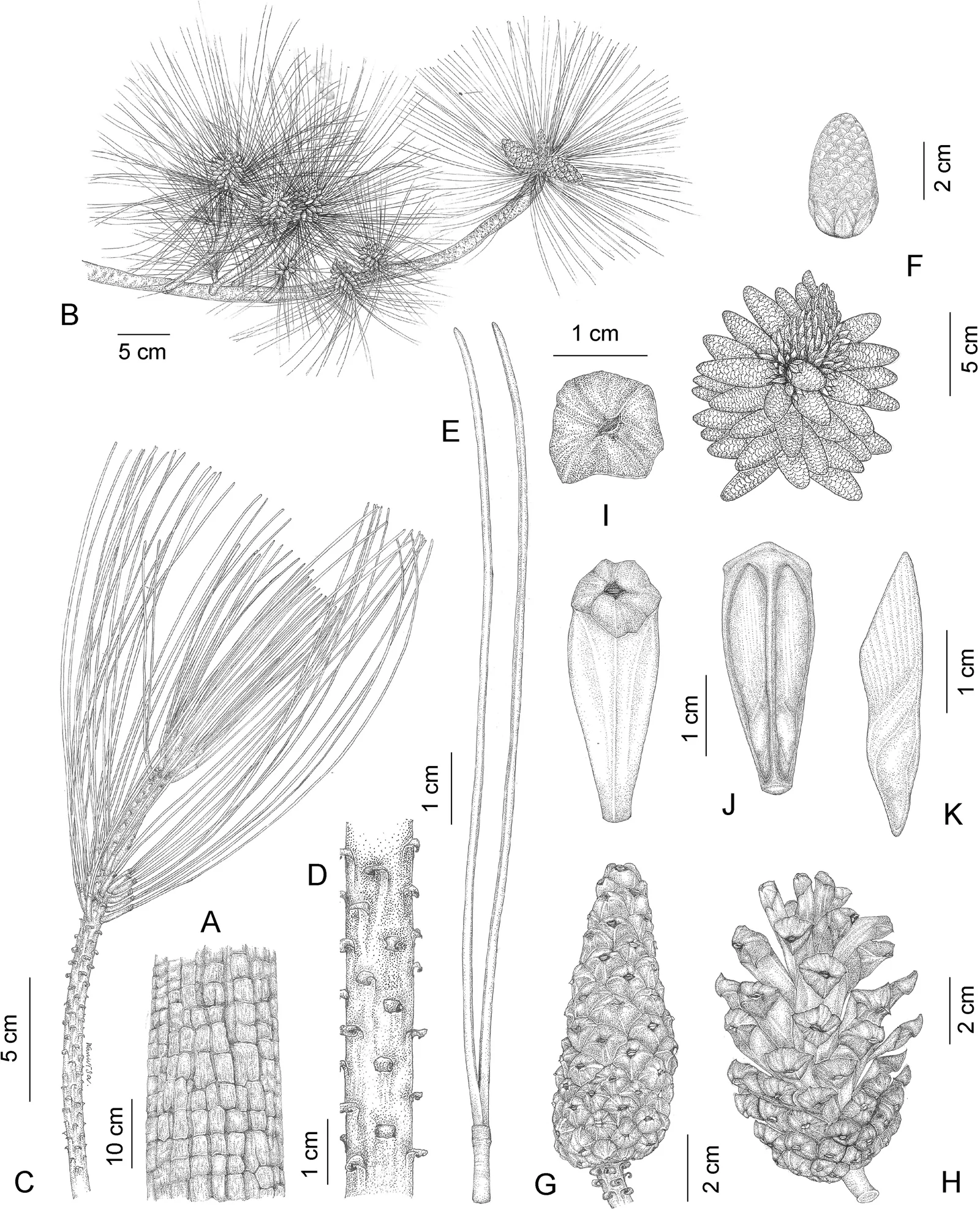
*Pinus latteri*. (A) Stem and outer bark. (B) Branchlets, leaves, pollen cones, and closed seed cone. (C) Branchlet and leaves. (D) Branchlet showing scars of fallen needle fascicles. (E) Needle fascicle. (F) Pollen cones. (G) Closed seed cone. (H) Open seed cone. (I) Seed scales. (J) Seed scale and winged seeds. (K) Winged seed. Image Source Credit: Wanwisa Bhuchaisri.

**Figure 7:**
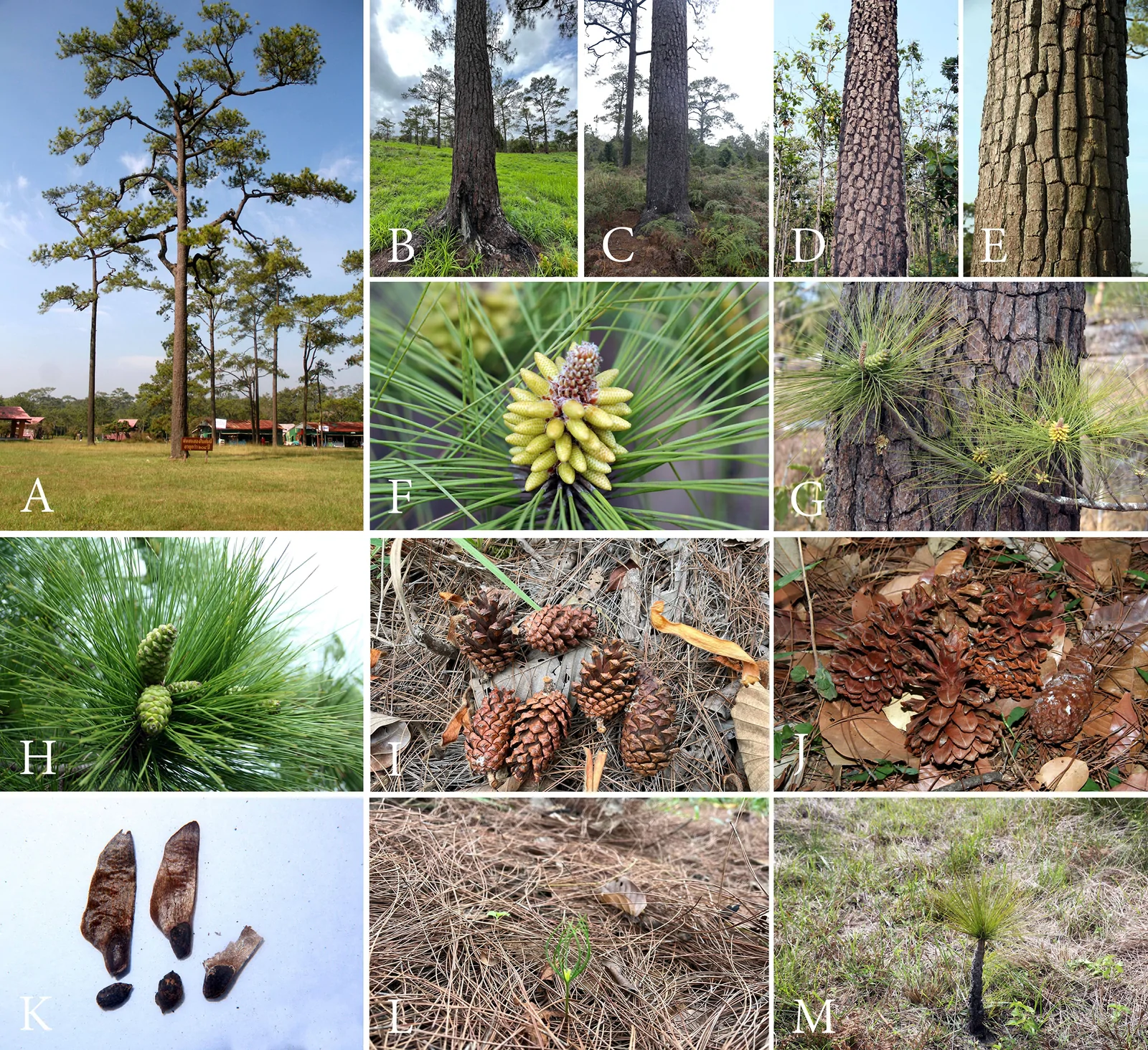
*Pinus latteri*. (A) Habit. (B–E) Stems and outer bark. (F) Branchlets, leaves, and pollen cones. (G) Outer bark, branchlets, leaves, pollen cones, and closed seed cones. (H) Branchlets, leaves, and closed seed cones. (I, J) Open seed cones. (K) Winged seeds (L) Seedling with several cotyledons. (M) Seedling with a grass stage. Image Source Credit: Chatchai Ngernsaengsaruay (A–J and L–M) and Pichet Chanton (K).

**Description.** Trees 10–35(–40) m tall; trunk usually straight, cylindrical, 50–310 cm GBH. ***Bark*** deeply fissured longitudinally and transversely, forming irregular, very thick, rough, slightly to distinctly raised plates, 2.5–5.5 cm thick, grayish brown to blackish brown. ***Branches*** spreading to ascending, usually crooked, crown irregular, broadly domed, umbrella-shaped, or flat-topped. ***Foliage branchlets*** slender, rough from leaf fascicle scars. ***Terminal buds*** cylindrical, 1.5–2 cm long; cataphylls reddish brown. ***Leaves*** in fascicles of 2, basal sheath persistent, reddish brown to brown, 1.2–2 cm × 1.5–2.5 mm; needles 14.5–28 cm × 1.2–1.7 mm, green, glabrous, rigid, straight, semicircular in transverse section, apex acuminate, margins minutely serrulate. ***Pollen cones*** clustered, spirally arranged at the base of new shoots, cylindrical, 1.6–2.5 × 0.7–0.8 cm, pale green when young, yellow to brownish yellow at maturity, brown when dry. ***Ovulate cones*** subglobose, green. ***Seed cones*** solitary or in clusters of 2–5, shortly pedunculate, spreading or reflexed, persistent; closed cones conical-ovoid, symmetrical or slightly asymmetrical, 5–10 × 2–4.5 cm (length/width ratio 2.0–2.9); open cones ovoid or broadly ovoid, 6–10 × 4–7 cm (length/width ratio 1.2–1.5); scales green when immature, lustrous, reddish brown at maturity, thick woody, more or less recurved when spreading, oblong, 2–3.5 × 1.3–1.5 cm; 4–5.5 mm thick at mid-length; apophyses prominently raised, more or less pyramidal, rhombic, pentagonal, or hexagonal, irregular in outline, distinctly transversely or radially ridged, central part not concave or shallowly concave; umbo small, elliptic in outline, slightly protruding, armed with a tiny deciduous spine or without. ***Seeds*** ellipsoid, 5.5–8 × 3.5–5 mm, reddish brown to dark brown; wing articulated to seed, 2–2.6 × 0.5–0.9 cm, membranous, persistent, 3–4.5 times as long as the seed. ***Seedlings*** with 9–10 or more cotyledons, with a grass stage (Figs. 6 and 7).

**Distribution.** Myanmar, China (Guangdong: Hainan Island; Guangxi), Vietnam, Laos, Cambodia, and Thailand (Fig. 4B).

**Distribution in Thailand. Northern:** Mae Hong Son (Pai, Tham Pla–Namtok Pha Suea), Chiang Mai (Doi Suthep-Pui, Doi Inthanon, Op Khan, Ban Wat Chan, Bo Luang, Chiang Dao: Ban Pa Lo, Fang, Mae Taeng), Chiang Rai (Khun Chae, Huan San, Wiang Pa Pao), Phayao [Chiang Kham: Ban Wang Tham], Lamphun (Doi Khun Tan, Li, Ban Hong: Doi Chang), Lampang (Doi Chong), Tak (Namtok Pha Charoen, Mae Sot), Phitsanulok (Phu Hin Rong Kla, Thung Non Son), Kamphaeng Phet (Khao Sanam Phriang); **North-Eastern:** Phetchabun (Nam Nao, Thung Salaeng Luang); Loei (Phu Kradueng, Phu Ruea, Phu Luang); **Eastern:** Chaiyaphum (Phu Khiao), Surin (Sangkha: Pa Son Nong Khu Forest Park), Si Sa Ket (Nam Kliang: Ban Nong Pha Naeng; Kanthararom: Ban Nong I Kwang), Ubon Ratchathani (Khong Chiam: Ban Ba Hai, Pha Cha Na Di); **South-Western:** Phetchaburi (Tha Yang: Khao Son Community Forest); **Central:** Suphan Buri (Phu Toei) (Fig. 4B).

**Figure 8:**
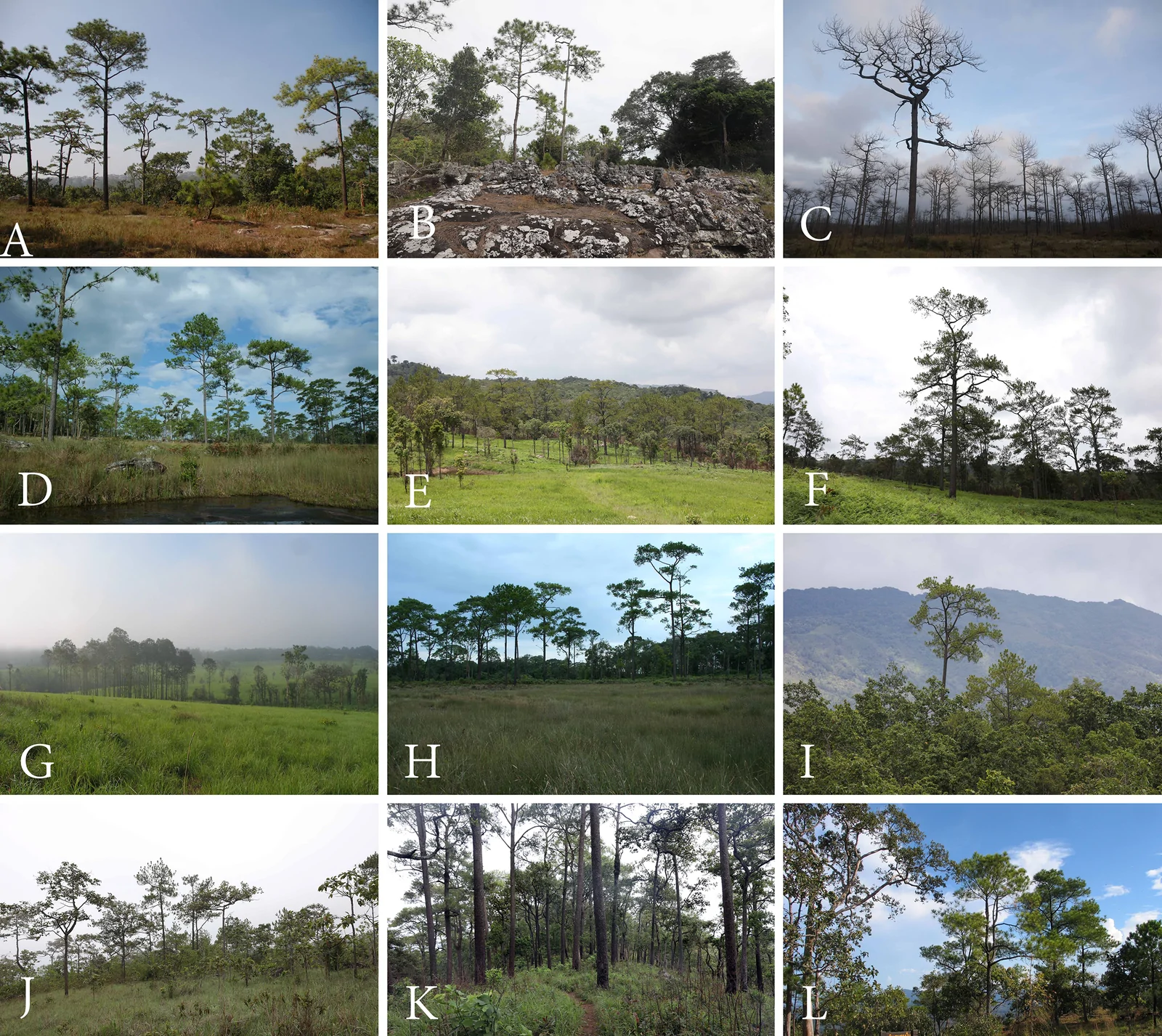
Habitats and ecology of *Pinus latteri* in Thailand. (A) Lower montane pine-oak forest at Phu Kradueng, Loei Province, at an elevation of ca. 1,200 m. a.m.s.l. (B) Lower montane pine-oak forest at Phu Hin Rong Kla, Phitsanulok Province, at an elevation of ca. 1,100 m. a.m.s.l. (C, D) Lower montane pine–oak forest (pine–oak savanna-like structure) at Phu Kradueng, Loei Province, at an elevation of ca. 1,200 m a.m.s.l. (E, F) Lower montane pine-oak forest (pine-oak savanna-like structure) at Phu Luang, Loei Province, at an elevation of ca. 1,250 m. a.m.s.l. (G) Pine-deciduous dipterocarp forest at Thung Salaeng Luang, Phetchabun Province, at an elevation of ca. 700 m a.m.s.l. (H) Pine-deciduous dipterocarp forest at Thung Non Son, Phitsanulok Province, at an elevation of ca. 950 m a.m.s.l. (I) Pine-deciduous dipterocarp forest at Mae Chaem, Chiang Mai Province, at an elevation of ca. 770 m a.m.s.l. (J) Pine-deciduous dipterocarp forest at Phu Ruea, Loei Province, at an elevation of ca. 1,200 m a.m.s.l. (K) Pine-deciduous dipterocarp forest at Phu Toei, Suphan Buri Province, at an elevation of ca. 750 m a.m.s.l. (L) Pine-deciduous dipterocarp forest at Pha Chana Dai, at an elevation of ca. 350 m a.m.s.l. Image Source Credit: Chatchai Ngernsaengsaruay.

**Habitat and ecology.** *Pinus latteri* is found in pine-deciduous dipterocarp forests and lower montane pine-oak forests, at elevations ranging from 70–1,350 m a.m.s.l. It can be found in association with *P. kesiya* at elevations of 800–1,350 m a.m.s.l. In Thailand, the lowest elevation record of *P. latteri* is from pine-deciduous dipterocarp forest at Khao Son Community Forest, Khao Ka Puk Subdistrict, Tha Yang District, Phetchaburi Province, at approximately 70 m a.m.s.l. Its highest elevation record in the same forest type is from Doi Inthanon National Park, Chiang Mai Province, at about 1,350 m a.m.s.l. This is consistent with the report by Santisuk (1997). Lower montane pine-oak forests in some localities may exhibit a pine (*P. latteri*)-oak savanna-like structure, such as at Phu Kradueng (ca. 1,200 m a.m.s.l.) and Khok Huai Toei, Phu Luang (1,230–1,250 m a.m.s.l.) (Fig. 8).

**Phenology.** Pollen cones January–March; ovulate cones March–April (–June); pollination during the dry season; fertilization following pollination; seed cones present throughout the year, requiring 2 years to mature.

**Conservation status.** *Pinus latteri* is distributed from Myanmar and China (Guangdong Province; Guangxi) to mainland Indo-China and Thailand. It has been recorded from numerous localities and has an EOO of 1,669,899.34 km^2^ and an AOO of 308 km^2^. In Thailand, it occurs naturally in many localities across five floristic regions, with an EOO of 338,610.06 km^2^ and an AOO of 176 km^2^. Although the species has a wide distribution, its populations are locally fragmented and some habitats are affected by deforestation and land-use change, resulting in declines in habitat quality. In addition, in some areas seed germination is limited, and seedlings that emerge often fail to establish or develop into mature individuals. We therefore assess its conservation status as Near Threatened (NT), following Nguyen et al. (2004), Farjon (2010), and Thomas (2013).

**Etymology.** *Pinus latteri* was named by the Rev. F. Mason in honor of Capt. Latter, the discoverer of the species, as all reliable information concerning the tree was obtained from him, beyond the previously vague information that a pine species occurred somewhere along the Salween River (Mason, 1849; Farjon, 2010).

**Vernacular names.** Kia plueak dam (เกี๊ยะเปลือกดำ) (Northern); Kia plueak na (เกี๊ยะเปลือกหนา) (Chiang Mai); Chuang (จ๋วง) (Northern, North-eastern); Chiang-sao (เชี้ยงเซา) (Karen-Mae Hong Son); Cho (โช) (Karen-Chaing Mai); Tai (ไต้) (Si Sa Ket, Ubon Ratchathani); Paek (แปก) (Loei, from the specimen *A. F. G. Kerr 8739*, Shan-Mae Hong Son); Son khao (สนเขา), **Son song bai** (**สนสองใบ**), Son hang ma (สนหางหมา) (Central); Saron (สะรอล) (Suai-Surin, from the specimen *A. F. G. Kerr 8263*); Shaja, tinshu (Burmese) (Farjon, 2010); Nan ya song (Chinese) (Fu, Li & Mill, 1999; Farjon, 2005, 2010); Thông nhựa, Thông hai lá (Vietnamese) (Nguyen et al., 2004); Laos pine (Silba, 1990), Tenasserim pine (Farjon, 2005, 2010), Two-needled pine (English).

**Uses.** Its uses are similar to those of *P. kesiya*.

**Notes**. *Pinus latteri* is closely related to *P. merkusii* but differs in having rigid needles, 1.2–1.7 mm diam. (*vs*. needles pliant, ca. 1 mm diam. in *P. merkusii*). The morphological characters of *P. merkusii* are based on Farjon (2010).

*Pinus latteri* was described by Mason (1849), who cited the type locality from [Myanmar], in provincia Amherst, in convalli fluvii Thoungyeen [in the valley of the Thoungyeen], collected by Latter (without collector number). However, no original material has been traced, and the whereabouts of the specimen remain unknown. No specimens have been located in any herbarium.

**Additional specimens examined. THAILAND. Northern: Mae Hong Son** [Locality unspecified, s.d., *Unknown s.n*. (CS [195370]); Tham Pla–Namtok Pha Suea National Park, seed cone, 11 Nov 2013, *C. Lakoet 0442* (BKF)]; **Chiang Mai** [Chiang Dao, 3 Mar 1957, *Khanchai 393* (BKF); Fang District, 27 Feb 1958, *T. Sørensen et al. 1729* (BKF); Bo Luang, seed cone, 13 Mar 1967, *E. N. Cooling 324* (K [K000554172]); ibid., ♂ cone, 13 Mar 1967, *E. N. Cooling 325* (K [K000554168]), *E. N. Cooling 326* (K [K005457247]); ibid., seedling with a grass stage, 14 Mar 1967, *E. N. Cooling 330* (K [K000554171]); Doi Suthep, ♂ cone, 21 Mar 1951, *T. Smitinand 159* (US [2036221]); ibid., 8 Feb 1957, *T. Smitinand 3774* (BKF); Doi Inthanon National Park, Mae Chaem District, ♂ cone & seed cone, 11 Feb 1998, *F. Konta et al. 4232* (BKF); Doi Inthanon, 2 Dec 1998, *M. Hara et al. 927* (BKF); ibid., ♂ cone & seed cone, 11 Feb 1998, *F. Konta & S. Khao-iem 10995* (BKF); Doi Inthanon National Park, Ban San Phatthana, Mae Chaem District, pine-deciduous dipterocarp forest, ca. 770 m a.m.s.l., seed cone, 24 May 2023, *C. Ngernsaengsaruay* personal observation with photos; Doi Inthanon National Park, Ban Mae Pa Ko, Chom Thong District, pine-deciduous dipterocarp forest, ca. 630 m a.m.s.l., 25 May 2023, *C. Ngernsaengsaruay* personal observation with photos; Mae Taeng District, lower montane pine-oak forest, 1,030–1,080 m a.m.s.l., 26 May 2023, *C. Ngernsaengsaruay* personal observation with photos]; **Chiang Rai** [Huai San, ♂ cone, 7 Mar 1912, *A. F. G. Kerr 2506* (K [K000554165]); between Mae Suai and Huai San, 5 Jan 1922, *J. F. Rock 1936* (US [1330139]); Wiang Pa Pao District, pine-deciduous dipterocarp forest, ca. 830 m a.m.s.l., 1 Feb 2013, *C. Ngernsaengsaruay* personal observation with photos; Khun Chae National Park, Wiang Pa Pao District, pine-deciduous dipterocarp forest, 920–930 m a.m.s.l., seed cone, 28 May 2023, *C. Ngernsaengsaruay* personal observation with photos]; **Phayao** [Ban Wang Tham, Chiang Kham District, 25 Jun 1954, *T. Smitinand 1649* (BKF)]; **Lamphun** [Doi Khun Tan National Park, Mae Tha District, ♂ cone, 28 Feb 1994, *J. F. Maxwell 94-278* (BKF, L [L.4178594]); Doi Chang, Ban Pa Pae, Pa Phlu Subdistrict, Ban Hong District, pine-deciduous dipterocarp forest, ca. 1,100 m a.m.s.l., seed cone, 22 Apr 2012, *C. Ngernsaengsaruay* personal observation with photos]; **Lampang** [Doi Chong National Park, ♀ cone & seed cone, Jun 1933, *I. Churuethai 2* (BKF)]; **Tak** [Mae Sot, seed cone, 15 Mar 1967, *E. N. Cooling 334* (K [K000554166]); Huai Sak Ngam Forest, Li District, s.d., *Saisaeng 7* (BKF)]; **Phitsanulok** [Lan Hin Taek, Phu Hin Rong Kla National Park, Nakhon Thai District, lower montane pine-oak forest, ca. 1,100 m a.m.s.l., ♀ cone & seed cone, 25 Apr 2007, *C. Ngernsaengsaruay* personal observation with photos; Thung Non Son, Thung Salaeng Luang National Park, Chom Phu Subdistrict, Noen Ma Prang District, pine-deciduous dipterocarp forest, ca. 950 m a.m.s.l., seed cone, 16 Oct 2007, *C. Ngernsaengsaruay* personal observation with photos]; **North-Eastern: Phetchabun** [Thung Salaeng Luang National Park, seed cone, 25 Jul 1973, *G. Murata et al. T-17134* (BKF, L [L.1183262]); Nong Mae Na–Thung Nang Phaya route, Thung Salaeng Luang National Park, Khao Ko District, pine-deciduous dipterocarp forest, 700–900 m a.m.s.l., seed cone, 29 Jan 2007, *C. Ngernsaengsaruay* personal observation with photos; Locality unspecified, 4 Jan 1960, *T. Smitinand 6288* (BKF)]; **Loei** [Phu Kradueng National Park, 12 Mar 1924, *A. F. G. Kerr 8739* (K [K000554200]); ibid., 21 Sep 1934, *T. Smitinand 1976* (BKF); ibid., 23 Jul 1948, *Din 61* (BKF); ibid., 29 Mar 1949, *D. Bunpheng 290* (BKF, BR [BR0000026050072V]); ibid., ♂ cone, 11 Feb 1952, *D. Bunpheng 479* (BKF); ibid., 9 Jul 1959, *T. Smitinand 5879* (BKF); ibid., 12 Jan 1960, *E. C. Abbe et al. 9465* (BKF, K [K000554164], NY [4278112]); ibid., 11 Sep 1963, *T. Smitinand & H. Sleumer 4780* (L [L.1183260]); ibid., 7 Nov 1970, *C. Charoenphol et al. 4696* (BKF); ibid., 11 Mar 1973, *T. Smitinand 11800* (BKF); ibid., 2 Nov 1984, *G. Murata et al. T-42788* (BKF); ibid., seed cone, 22 Jun 2015, *S. Tagane et al. T4519* (BKF); ibid., seedling with a grass stage, 8 Jun 2020, *T. Thananthaisong et al. 14* (BKF); Phu Kradueng National Park, lower montane pine-oak forest, ca. 1,200 m a.m.s.l., seed cone, 27 Oct 2013 and 13 Nov 2021, *C. Ngernsaengsaruay* personal observation with photos; Phu Ruea National Park, 19 Jan 2010, *M. S. Nuraliev & I. A. Savinov 421* (MW [MW0732137]); Phu Ruea National Park, pine-deciduous dipterocarp forest, 1,170–1,200 m a.m.s.l., seed cone, 12 Jun 2023, *C. Ngernsaengsaruay* personal observation with photos; Khok Huai Toei, Phu Luang Wildlife Sanctuary, lower montane pine-oak forest, 1,230–1,250 m a.m.s.l., seed cone, 11 Jun 2023, *C. Ngernsaengsaruay* personal observation with photos]; **Eastern: Surin** [Sangkha District, 12 Jan 1924, *A. F. G. Kerr 8263* (K [K000554202])]; **Si Sa Ket** [Nong I Kwang, Kanthararom District, 13 Jul 1951, *Unreadable 123* (BKF [10759]); Ban Nong Pha Naeng, Nam Kliang District, ♂ cone & seed cone, 11 Mar 1967, *S. Phusomsaeng 50* (BKF, K [K000554203], L [L.1183257])]; **Ubon Ratchathani** [Ban Ba Hai, Khong Chiam District, seed cone, 20 Aug 1986, *C. Niyomdham 1238* (BKF); Pha Cha Na Di, Dong Na Tham Forest, Pha Taem National Park, Na Pho Klang Subdistrict, Khong Chiam District, pine-deciduous dipterocarp forest, ca. 350 m a.m.s.l., 12 Nov 2005, C. *Ngernsaengsaruay* personal observation with photos]; **South-Western: Phetchaburi** [Tha Yang District, seed cone, 16 Feb 2006, *D. J. Middleton et al. 3709* (BKF, E [E00261729], K [K000577002], L [L.4178643]); Khao Son Community Forest, Khao Ka Puk Subdistrict, Tha Yang District, pine-deciduous dipterocarp forest, ca. 70 m a.m.s.l., 7 Jul 2023, *C. Ngernsaengsaruay* personal observation with photos]; **Central: Suphan Buri** [Phu Toei National Park, seed cone, 29 Jun 2001, *T. Wongprasert 016-14* (BKF); Phu Toei National Park, Dan Chang District, pine-deciduous dipterocarp forest, ca. 750 m a.m.s.l., seed cone, 28 Jul 2023, *C. Ngernsaengsaruay* personal observation with photos].

A comparison of the morphological characteristics of *P. kesiya* and *P. latteri* in Thailand with those reported in previous studies is presented in Table 1.

**Table 1:** Comparison of the morphological characters of *Pinus* in Thailand with those reported in previous studies from other countries. The morphological characters of *P. kesiya* and *P. latteri* from other countries were obtained from following sources: [1] Farjon (2010), [2] Farjon (2005), [3] Li (1997), [4] Fu, Li & Mill (1999), [5] Phan et al. (2017), and [6] Averyanov et al. (2014).

Characters	From the author’s observations		Previous studies	
	*Pinus kesiya*	*Pinus latteri*	*Pinus kesiya*	*Pinus latteri*
Bark	Fissured forming irregular, flat, rough plates, 0.3–1 cm thick, grayish brown or reddish brown	Deeply fissured longitudinally and transversely, forming irregular, very thick, rough, slightly to distinctly raised plates, 2.5–5.5 cm thick, grayish brown to blackish brown	Deeply fissured forming irregular plates, rough and flaking, brown weathering grey-brown^[1]^; brown, irregularly flaking^[4]^ when old. Bark brown, thick, irregularly reticulate and deeply fissured, flaking in thick pieces^[5]^; orange-brown, later dull gray-brown, irregularly flaking^[6]^	Thick and scaly, rough, breaking into numerous small, dark grey plates^[1]^; bark gray brown, thick, scaly^[5]^; rough, gray-brown, deeply fissured^[6]^
New foliage shoots	Not observed	Not observed	Multi-nodal^[1]^; bi- or multi-nodal (two or more nodes each year)^[3], [4]^;	Uni-nodal^[1]^
Leaves	Needles in fascicles of 3	Needles in fascicles of 2	Needles in fascicles of 3^[1], [2], [3], [4], [5], [6]^	Needles in fascicles of 2^[1], [2], [3], [4], [5], [6]^
Basal sheath length (cm)	0.75–1.5	1.2–2	1.5–2^[1], [2]^; 1–2^[4], [6]^; 1.5–1.8^[5]^	1.5–2^[1], [2]^; 1–2^[4]^; ca. 2^[5]^
Basal sheath width (mm)	1–1.5	1.5–2.5	Not recorded	Not recorded
Needle form	Lax, drooping	Rigid, straight	Lax, drooping (very lax or flexible)^[1]^; pliant^[4]^; flexible^[5]^; rigid^[6]^	Rigid, straight^[1]^; rigid^[6]^
Needle shape in transverse section	Triangular	Semicircular	Triangular^[4], [6]^	Semicircular^[1], [2], [4], [6]^
Needle length (cm)	(10–)13–25	14.5–28	(10–)12–22(–25)^[1]^; 12–25^[2]^; 10–22^[4], [6]^; 10–24^[5]^	15–25(–27)^[1]^; 15–25^[2]^; 9–25^[3]^; 15–27^[4]^; 19–32^[5]^; 20–27^[6]^
Needle width (mm)	0.7–1	1.2–1.7	0.5–0.7(–1)^[1]^; 0.5–0.7^[2]^; 0.5–0.8^[3]^; 0.7–1^[4], [5]^	ca. 1.5^[1], [2], [4], [5]^
Pollen cone shape	Cylindrical	Cylindrical	Short cylindrical^[1]^; ovoid, later fusiform^[6]^	Cylindrical^[1], [5]^; ovoid^[6]^
Pollen cone length (cm)	1.6–2.5	1.6–2.5	Not recorded	2–3 [1], 3–3.5 [5]
Pollen cone width (mm)	5–9	7–8	Not recorded	Not recorded
Seed cone shape (closed)	Conical-ovoid, sometimes broadly conical-ovoid, symmetrical or slightly asymmetrical	Conical-ovoid, symmetrical or slightly asymmetrical	Narrowly ovoid-conical, symmetrical or slightly non-symmetrical^[1]^; void to ovoid-conical^[5]^; ovoid to conic^[6]^	Ovoid-conical or oblong-conical^[1], [2]^; conical or ovoid-cylindric^[4], [5]^; ovoid to broadly conic^[6]^
Seed cone length (closed) (cm)	5–8 (length/width ratio 1.1–1.9)	5–10 (length/width ratio 2.0–2.9)	4.5–7(–8)^[1]^; 5–6^[4]^; 5–7.5^[5]^; 4–6^[6]^	(5–)6–10(–13)^[1]^; 6–13^[2]^; 5–10^[4]^; 7–13^[5]^; 6–12^[6]^
Seed cone width (closed) (cm)	3–5	2–4.5	ca. 3.5^[4]^; 4.5–6^[5]^; 3–3.5^[6]^	5–7^[5]^; 5–8^[6]^
Seed cone shape (open)	Broadly ovoid or subglobose	Ovoid or broadly ovoid	Ovoid to subglobose^[1]^	Broadly ovoid with a flat base^[1]^
Seed cone length (open) (cm)	5–8 (length/width ratio 1.0–1.4)	6–10 (length/width ratio 1.2–1.5)	4.5–7^[2]^	Not recorded
Seed cone width (open) (cm)	5–6.5	4–7	3–4(–5.5)^[1]^; 3–4^[2]^	4–9^[1]^
Seed scale length (cm)	2–3.5	2–3.5	2.5–3^[1], [4], [5], [6]^	ca. 3^[1], [4]^; twice as long as wide^[1], [2]^; 2.9–3.5^[5]^
Seed scale width (cm)	1–1.5	1.3–1.5	1–1.5^[1], [4], [5]^	1.2–1.5^[1], [4], [5]^
Seed scale thickness at mid-length (mm)	2–3.5, thin woody	4–5.5, thick woody	Not recorded	Not recorded
Apophysis	Prominently raised, more or less pyramidal, rhombic, irregular in outline, distinctly transversely or radially ridged, central part shallowly concave	Prominently raised, more or less pyramidal, rhombic, pentagonal, or hexagonal, irregular in outline, distinctly transversely or radially ridged, central part not concave or shallowly concave	Prominently raised, more or less pyramidal, irregularly rhombic in outline, transversely keeled^[1], [2]^; ± pyramidal, obviously transversely ridged^[4]^; rhombic, ca. 0.8 × 0.8 cm, obviously transversal ridges^[5]^; pyramidal, transversely ridged^[6]^	Rhombic or unequally pentagonal in outline, raised and strongly transversely keeled, radially striated or Ridged^[1], [2]^; apophyses subrhombic or pentagonal-rhombic, slightly swollen, shiny, slightly recurved toward apex, flat toward base, obviously radially ridged^[4]^; rhombic, ca. 1.3 × 1.3 cm, slightly swollen and recurved toward apex, flat toward base, obviously radially ridged^[5]^; flat and a prominent transverse keel^[6]^
Umbo	Small, usually sunken, elliptic in outline, sometimes slightly protruding, armed with a tiny recurved deciduous spine	Small, elliptic in outline, slightly protruding, armed with a tiny deciduous spine or without	Small, ellipsoid or at least protruding, armed with a very small, persistent prickle^[1], [2]^; small, ellipsoid, slightly protruded into a tiny recurved spine^[4]^; small, transversally ellipsoid, slightly protruded into a stout, short (less than 1 mm), tiny recurved spine^[5]^; small, shortly apiculate^[6]^	Dorsal, more or less central, slightly sunken or flat to obtuse, unarmed^[1], [2]^; usually slightly sunken^[4]^; small, transversally ellipsoid, slightly protruded into a stout, short (less than 1 mm) spine^[5]^
Seed shape	Ellipsoid	Ellipsoid	Ellipsoid^[1], [4], [5]^	Ellipsoid to obovoid^[1]^; ellipsoid-ovoid^[4], [5]^
Seed length (mm)	6–8.5	5.5–8	5–7(–8)^[1]^; 5–6^[4], [6]^; ca. 7^[5]^	5–8^[1], [2], [4]^; 6–9^[5]^
Seed width (mm)	4–5	3.5–5	3–4^[4]^; 5^[5]^; 3.5 (thick)^[5]^	ca. 4^[1], [4]^; 3.5–4^[5]^
Seed color	Reddish brown to dark brown	Reddish brown to dark brown	Dark brown to nearly black^[1]^; black-brown^[4], [6]^; brownish^[5]^	Grey-brown^[1], [2], [4], [5]^
Seed wing length (cm)	1.1–2.5 (1.5–3 times as long as the seed)	2–2.6 (3–4.5 times as long as the seed)	1.5–2^[1]^; 1.5–2.5^[5]^; 2.2–3.2 (including seed)^[5]^	1.5–2^[1], [2]^; not more than three times as long as the seeds^[1]^; 1.7–2^[4]^; 2.2–2.7 (including seed)^[5]^
Seed wing width (mm)	6–8	5–9	6–8^[1]^; ca. 6.5^[5]^	ca. 8^[5]^
Seedling grass stage	Without a grass stage	With a grass stage	Not recorded	With a grass stage^[1], [2], [3]^

### Anatomical study

#### Leaf anatomy of Thai *Pinus* species

**Figure 9:**
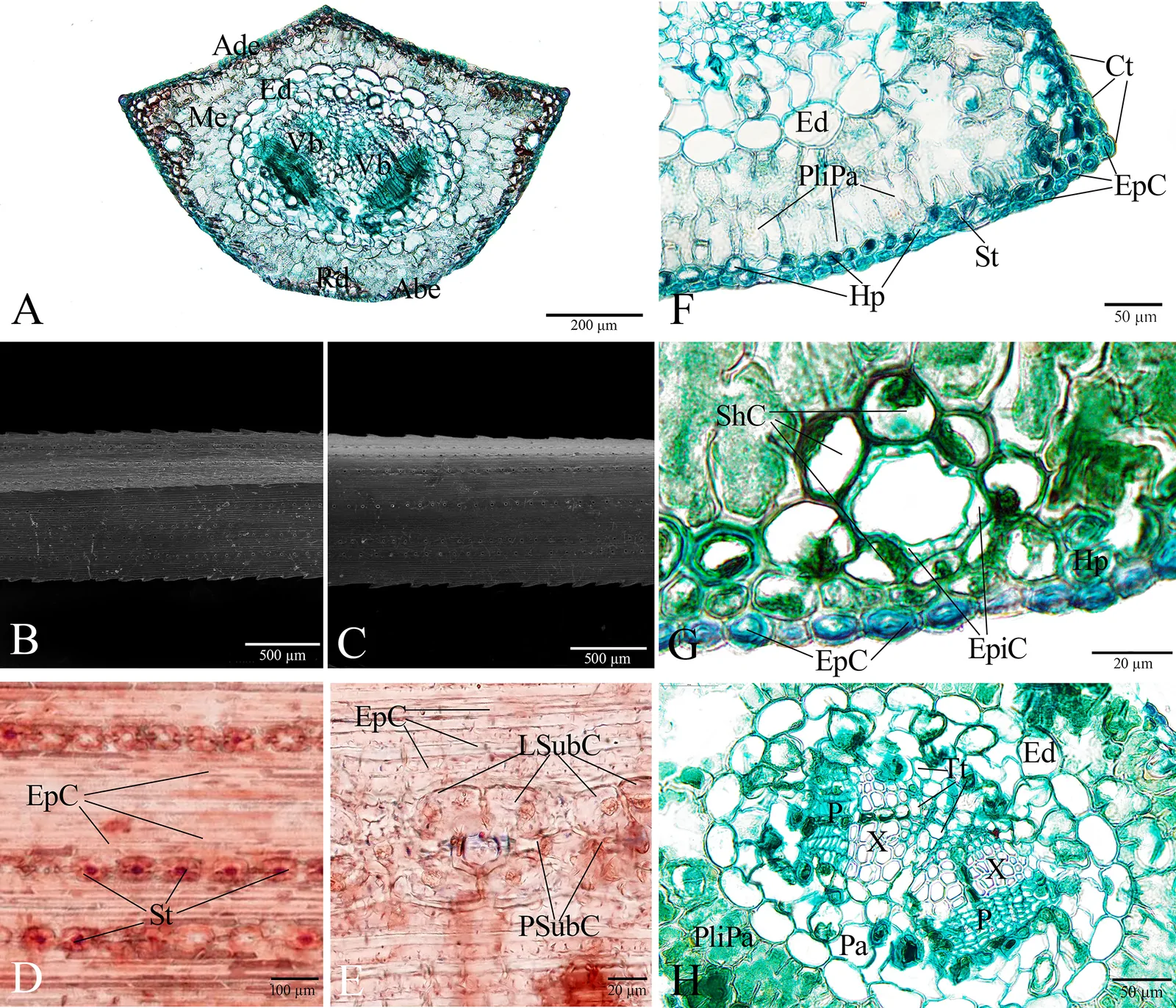
Leaf anatomy of *Pinus kesiya*. (A, F−H) Transverse section. (B) Adaxial epidermis (under SEM). (C) Abaxial epidermis (under SEM). (D, E) Abaxial epidermis (under LM). Abe, abaxial epidermis; Ade, adaxial epidermis; Ct, cuticle; EpiC, epithelial cell; Ed, endodermis; EpC, epidermal cells; Hp, hypodermis; LSubC, lateral subsidiary cells; Me, Mesophyll; P, phloem; Pa, parenchyma; PliPa, plicate parenchyma; PSubC, polar subsidiary cells; Rd, resin duct; ShC, sheath cell; St, stoma; Tt, transfusion tissue; Vb, vascular bundle; X, xylem. Image Source Credit: Pichet Chanton and Chatchai Ngernsaengsaruay.

**Figure 10:**
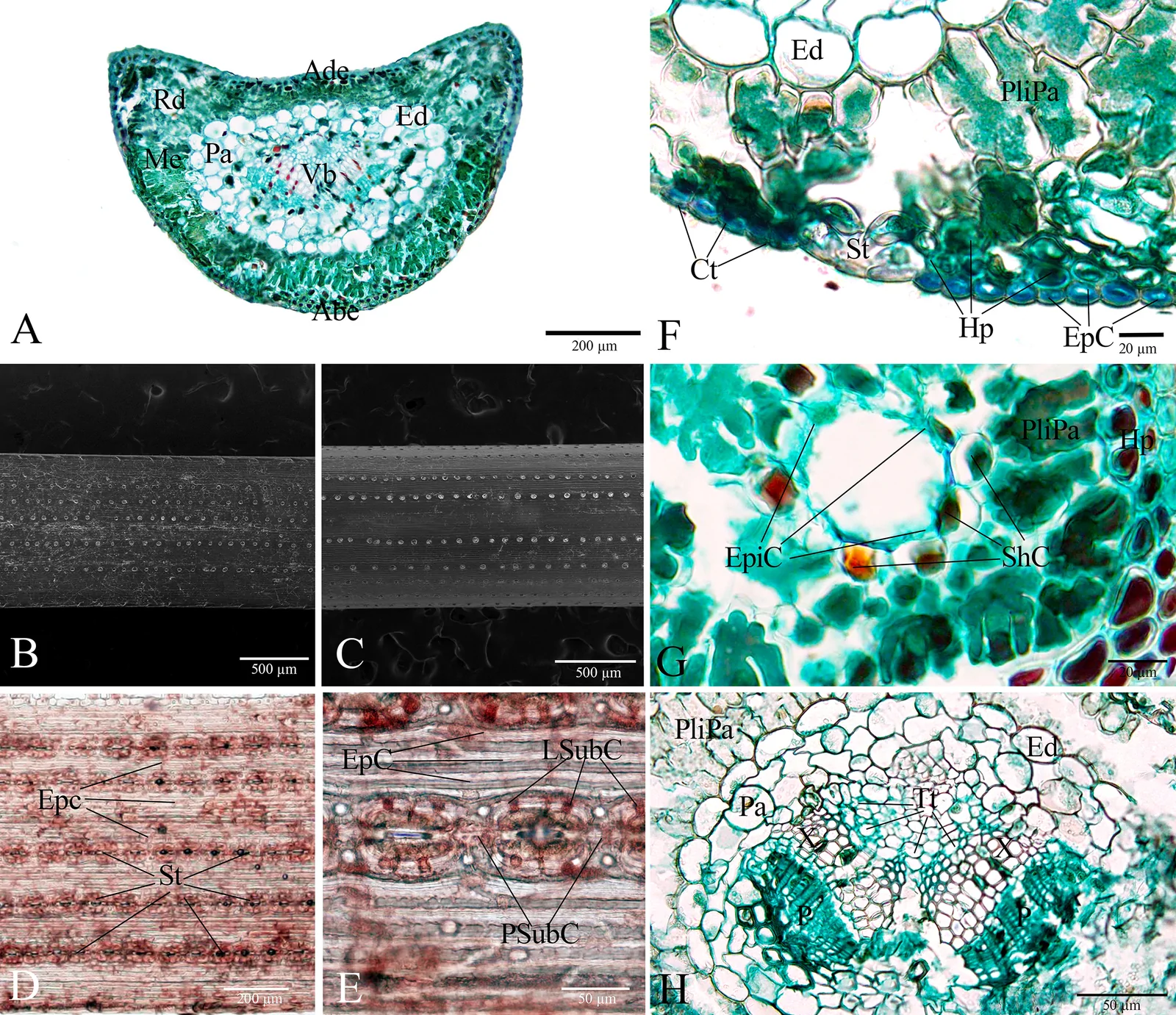
Leaf anatomy of *Pinus latteri*. (A, F−H) Transverse section. (B) Adaxial epidermis (under SEM). (C) Abaxial epidermis (under SEM). (D, E) Abaxial epidermis (under LM). Abe, abaxial epidermis; Ade, adaxial epidermis; Ct, cuticle; EpiC, epithelial cell; Ed, endodermis; EpC, epidermal cells; Hp, hypodermis; LSubC, lateral subsidiary cells; Me, Mesophyll; P, phloem; Pa, parenchyma; PliPa, plicate parenchyma; PSubC, polar subsidiary cells; Rd, resin duct; ShC, sheath cell; St, stoma; Tt, transfusion tissue; Vb, vascular bundle; X, xylem. Image Source Credit: Pichet Chanton and Chatchai Ngernsaengsaruay.

In transverse section, the needles appear triangular in the examined specimens of in *P. kesiya* and semicircular in *P. latteri*. In both species, the abaxial surface is distinctly curved, forming part of the circular outline. Although differences in needle transverse shape were observed, both species share the general anatomical features typical of the genus, including differentiation into dermal tissues (epidermis and hypodermis), mesophyll, and a vascular region containing two vascular bundles (Figs. 9 and 10).

The epidermis is single-layered and composed of thick-walled sclerenchymatous cells covered externally by a thick cuticle. The hypodermis, located beneath the epidermis, is one to two layers thick and also consists of thick-walled sclerenchymatous cells. The epidermal cells between the stomatal rows are elongated and rectangular, with wavy longitudinal anticlinal walls. Stomata are distributed on both leaf surfaces and arranged in 10–18 distinct rows. They are haplocheilic and sunken, representing a xerophytic adaptation that may reduce water loss (Figs. 9 and 10). According to Esau (1977), haplocheilic stomatal development is characterized by the formation of guard cells from a single protodermal initial, whereas subsidiary cells arise from adjacent epidermal cells. In both species, the stomatal complex consists of two guard cells, two polar subsidiary cells (one at each pole), and four lateral subsidiary cells (two on each side). The polar subsidiary cells are smaller than the lateral ones and are generally shorter than the intervening epidermal cells.

The mesophyll is undifferentiated, lacking separation into palisade and spongy parenchyma. It is monomorphic and composed of thin-walled parenchymatous cells containing numerous chloroplasts. Characteristically, the mesophyll contains plicate parenchyma cells. Resin ducts are present within the mesophyll. Each resin duct consists of two cell types: thick-walled sheath cells and thin-walled epithelial cells (Figs. 9 and 10).

Resin ducts in the needles occur in two positions: medial and external. Medial resin ducts are located in the mesophyll between the endodermis and hypodermis, whereas external resin ducts are in contact with the hypodermis. Variation in the number and position of resin ducts was observed between the examined specimens of *P. kesiya* and *P. latteri*. *Pinus kesiya* in the examined specimens has 3–4 external resin ducts, with two lateral and one or two on the abaxial surface. In contrast, *P. latteri* in the examined specimens has 2–3 medial resin ducts, consisting of two lateral, or two lateral and one on the abaxial surface.

A single continuous layer of endodermal cells is located between the plicate parenchyma and the transfusion tissue surrounding the central vascular bundles. The endodermal cells vary in size but have uniformly thickened walls. Two vascular bundles are embedded within the transfusion tissue, which is composed of transfusion parenchyma and transfusion tracheids. Within each vascular bundle, the phloem is located on the abaxial side, whereas the xylem is positioned on the adaxial side (Figs. 9 and 10).

A comparison of leaf anatomical characters of *Pinu*s species in Thailand is provided in Table 2.

**Table 2:** Comparison of leaf anatomical characters of *Pinus* species in Thailand.

Characteristics	From the author’s observations	
	*Pinus kesiya*	*Pinus latteri*
Needle shape in transverse section	Triangular	Semicircular
Cuticular wax thickness on both leaf surfaces (μM)	1.74–2.97 (2.37 ± 0.34)	0.94–3.23 (2.05 ± 0.53)
Number of epidermal cell layer on both leaf surfaces	One-layered	One-layered
Epidermal layer thickness on both leaf surfaces (μM)	7.23–14.68 (10.44 ± 2.04)	5.61–19.62 (11.30 ± 2.74)
Epidermal cell shape on both leaf surfaces	Broadly elliptic or elliptic	Broadly elliptic or elliptic
Stomatal types	Haplocheilic, sunken	Haplocheilic, sunken
Stomatal density per mm^2^	40–67 (52.53 ± 7.98) in 4–5 stomatal rows	45–65 (56.17 ± 5.77) in 4–5 stomatal rows
Stomatal shape	Broadly elliptic or elliptic	Broadly elliptic or elliptic
Stomatal length (μM)	47.46–77.04 (62.54 ± 9.04)	59.26–95.84 (74.93 ± 10.27)
Stomatal width (μM)	43.75–61.04 (53.22 ± 5.53)	47.48–68.35 (57.71 ± 5.75)
Number of subsidiary cells	Six [two polar subsidiary cells (one at each pole), and four lateral subsidiary cells (two on each side)]	Six [two polar subsidiary cells (one at each pole), and four lateral subsidiary cells (two on each side)]
Number of hypodermal cell layer on both leaf surfaces	One to three-layered	One to three-layered
Hypodermal layer thickness on the adaxial leaf surface (μM)	10.66–20.37 (14.69 ± 2.48)	10.28–26.74 (18.45 ± 6.97)
Hypodermal layer thickness on the abaxial leaf surface (μM)	9.19–29.63 (16.65 ± 4.66)	11.30–37.95 (20.00 ± 6.62)
Hypodermal cell shape on both leaf surfaces	Broadly elliptic or elliptic	Broadly elliptic or elliptic
Hypodermal cell length on both leaf surfaces (μM)	6.59–16.03 (11.41 ± 2.17)	9.59–20.54 (13.75 ± 3.07)
Hypodermal cell width on both leaf surfaces (μM)	11.34–20.28 (15.24 ± 2.25)	4.11–12.93 (8.43 ± 2.15)
Number and position of resin ducts	3–4 (3.43 ± 0.50); External: 3 (two lateral and one on abaxial surface); 4 (two lateral and two on abaxial surface)	2–3 (2.40 ± 0.50); Medial: 2 (two lateral); 3 (two lateral and one on abaxial surface)
Resin duct shape	Broadly elliptic, circular, or subcircular	Broadly elliptic, circular, or subcircular
Resin duct length (μM)	46.82–83.87 (62.68 ± 10.64)	51.58–85.87 (67.37 ± 10.32)
Resin duct width (μM)	42.69–92.67 (63.44 ± 11.74)	56.23–84.06 (69.91 ± 8.70)
Central cylinder shape	Broadly elliptic or subcircular	Broadly elliptic
Central cylinder length (μM)	254.84–426.39 (318.04 ± 47.08)	300.53–388.74 (349.29 ± 25.17)
Central cylinder width (μM)	348.86–498.30 (417.94 ± 50.89)	501.23–546.39 (528.61 ± 14.31)
Endodermal cell shape	Elliptic or broadly elliptic	Elliptic, broadly elliptic, or subcircular
Endodermal cell length (μM)	33.70–54.68 (41.51 ± 5.87)	32.76–77.84 (53.73 ± 10.47)
Endodermal cell width (μM)	17.16–33.63 (26.73 ± 3.55)	26.96–61.56 (47.48 ± 8.63)
Stelar portion length (μM)	212.50–368.86 (287.52 ± 49.23)	277.67–300.03 (290.42 ± 6.51)
Stelar portion width (μM)	298.65–434.20 (353.62 ± 38.10)	420.87–476.84 (446.04 ± 15.62)

### Palynological study

#### Pollen morphology of Thai *Pinus* species

**Figure 11:**
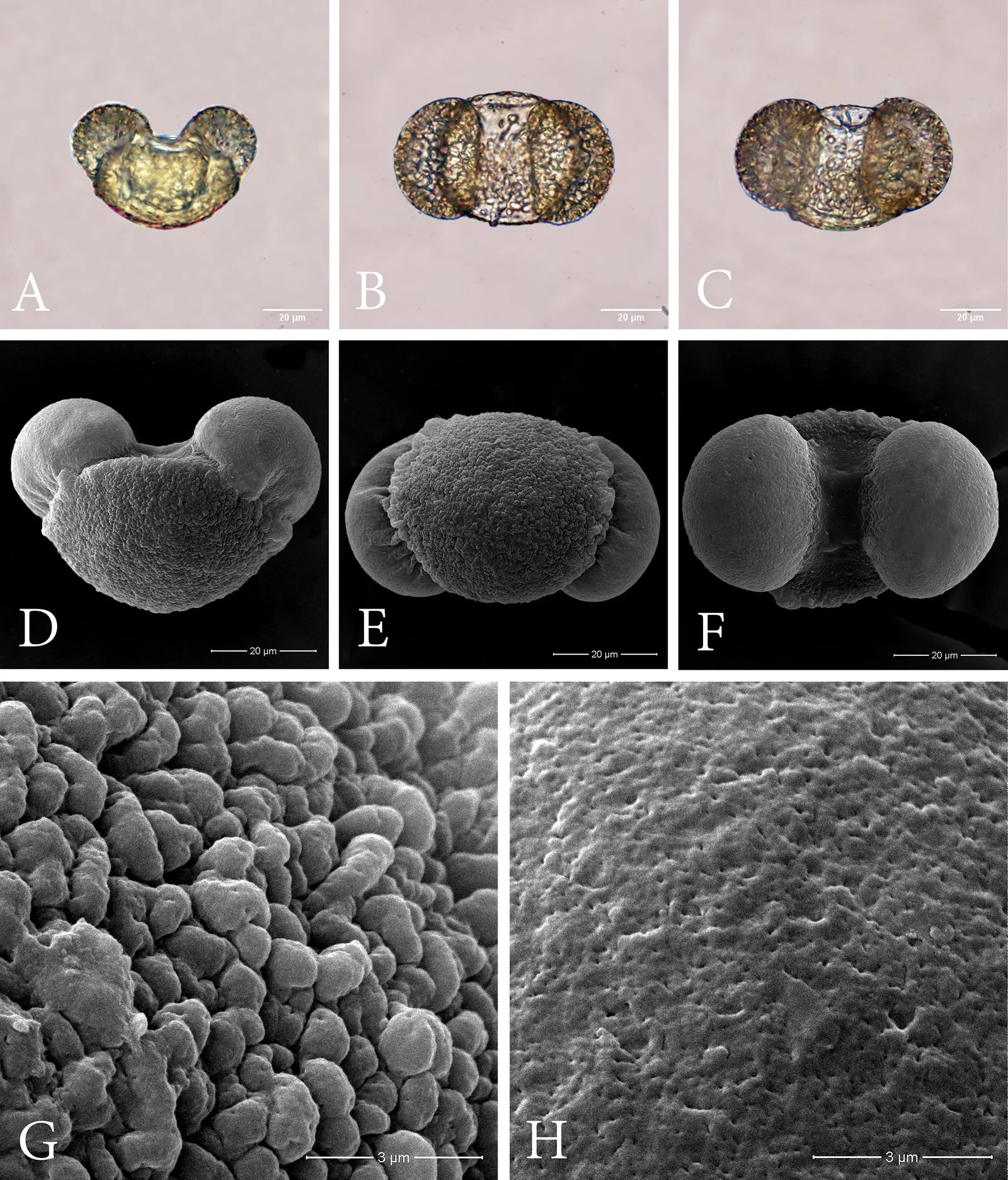
LM and SEM micrographs of pollen grains of *Pinus kesiya*. (A and D) Bisaccate pollen in equatorial view. (B and E) *Corpus* in polar view. (C and F) Sacci and aperture in polar view. (G) Exine sculpturing on the *corpus*. (H) Exine sculpturing on the sacci. (A–C under LM; D–H under SEM). Image Source Credit: Pichet Chanton (under LM); Chatchai Ngernsaengsaruay and Pichet Chanton (under SEM).

**Figure 12:**
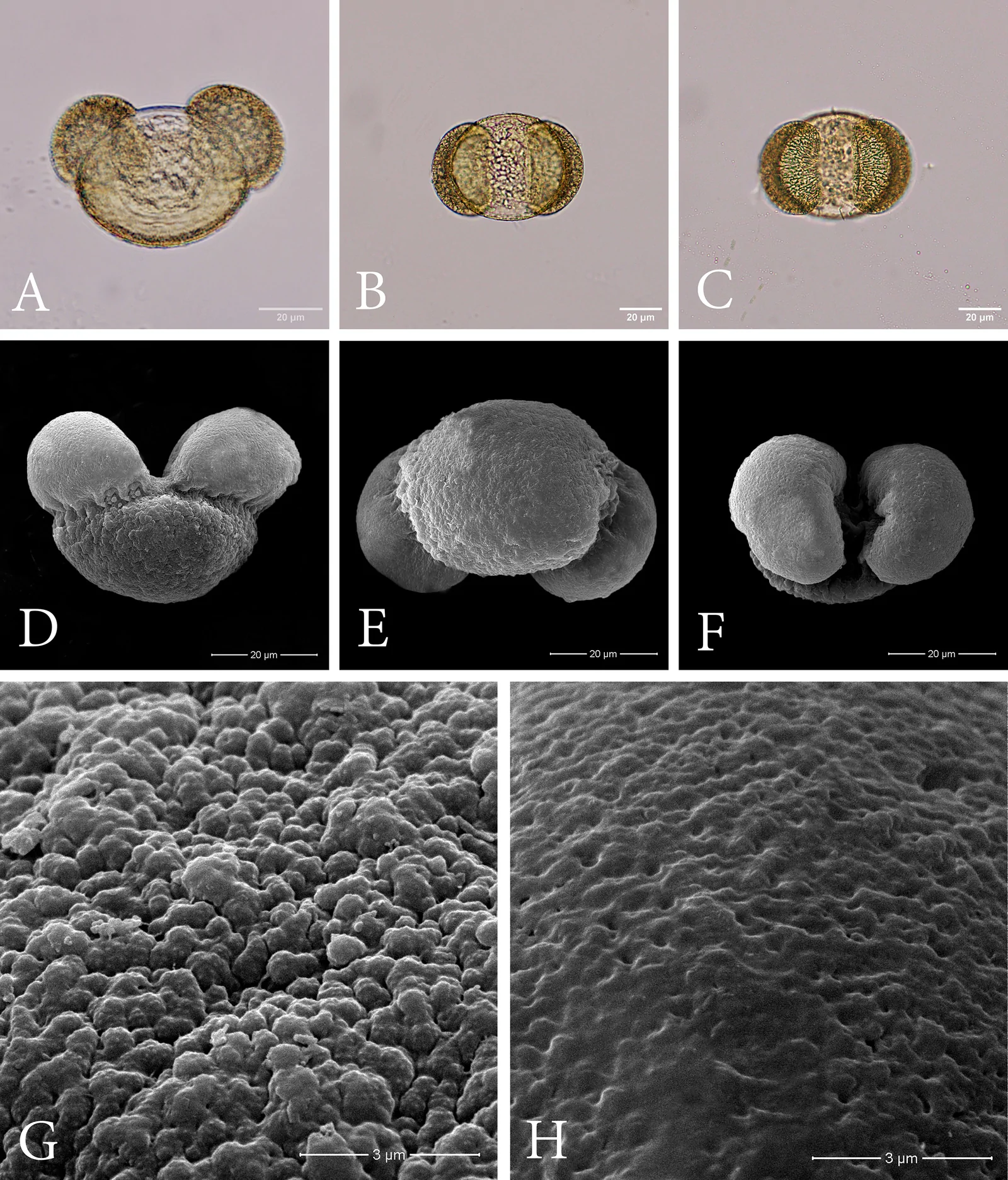
LM and SEM micrographs of pollen grains of *Pinus latteri*. (A and D) Bisaccate pollen in equatorial view. (B and E) *Corpus* in polar view. (C and F) Sacci and aperture in polar view. (G) Exine sculpturing on the *corpus*. (H) Exine sculpturing on the sacci. (A–C under LM; D–H under SEM). Image Source Credit: Pichet Chanton (under LM); Chatchai Ngernsaengsaruay and Pichet Chanton (under SEM).

The pollen grains of two Thai species of *Pinus* are monads and bisaccate. A monosulcate aperture is present on the distal pole between the sacci. The pollen shape ranges from oblate and suboblate to oblate-spheroidal, with a *corpus* length/width ratio of 0.50 to 0.99. The overall pollen size, including the sacci (air sacs), is medium to large, ranging from 44.00 to 74.90 µM. In polar view, the pollen length including sacci ranges from 32.17 to 55.69 µM, whereas in equatorial view, the pollen width including sacci ranges from 44.00 to 74.90 µM. The *corpus* (pollen body) ranges from 20.14 to 41.34 × 28.48 to 53.37 µM, and the sacci range from 16.83 to 31.61 × 17.33 to 31.56 µM. The exine thickness ranges from 1.16 to 4.04 µM. Exine sculpturing of the *corpus* is granulate, whereas that of the sacci is perforate. The pollen morphology of *P. kesiya* and *P. latteri* is highly similar. Although the pollen grains of *P. kesiya* tend to be slightly larger than those of *P. latteri*, their size ranges show substantial overlap. Therefore, pollen morphological characters are insufficient for species delimitation (Figs. 11 and 12).

A comparison of pollen morphology among *Pinus* species in Thailand is provided in Table 3.

**Table 3:** Pollen morphological comparison of Thai *Pinus* species.

Characters	Species	
	*Pinus kesiya*	*Pinus latteri*
Pollen length with sacci (µM)	36.70–55.69 (41.45 ± 3.81)	32.17–49.98 (40.94 ± 4.39)
Pollen width with sacci (µM)	52.78–74.90 (65.43 ± 4.24)	44.00–69.73 (56.46 ± 7.59)
Pollen size	Large	Medium to large
*Corpus* length (µM)	26.70–41.34 (32.95 ± 3.58)	20.14–36.75 (27.78 ± 4.37)
*Corpus* width (µM)	37.49–53.37 (44.43 ± 4.16)	28.48–47.69 (38.28 ± 4.80)
*Corpus* length/width ratio	0.50–0.98	0.53–0.99
Pollen shape (based on *corpus* length/width ratio)	Oblate, suboblate, oblate spheroidal	Oblate, suboblate, oblate spheroidal
Saccus length (µM)	19.65–29.43 (24.65 ± 2.35)	16.83–31.61 (22.87 ± 3.52)
Saccus width (µM)	19.60–29.73 (24.91 ± 2.65)	17.33–31.56 (24.30 ± 3.84)
Distance between sacci (µM)	5.56–23.50 (13.56 ± 4.53)	6.63–15.75 (9.48 ± 2.29)
Exine thickness (µM)	1.16–4.04 (2.81 ± 0.67)	2.02–3.93 (3.00 ± 0.52)
Exine sculpturing of the *corpus*	Granulate	Granulate
Exine sculpturing of the sacci	Perforate	Perforate

## Discussion

*Pinus* subgenus *Pinus* is characterized by decurrent pulvini; leaves containing two vascular bundles with resin ducts in variable positions; a persistent (rarely deciduous) fascicle sheath; and seeds bearing an articulate, rarely adnate, wing. Despite sharing these diagnostic features at the subgenus level, the two species are placed in different subsections. Subsection *Pinus* is characterized by multi-nodal shoots and buds that are slightly resinous or rarely non-resinous. Leaves are borne in fascicles of two, or of two and three, and exclusively three in *P. kesiya*. Seed cones are deciduous or persistent for a few years but always open soon, measuring 2–10(–12) cm long. The seed scales are thinly woody and rigid. The umbo is unarmed or bears a minute, deciduous or sometimes persistent prickle (*e.g*., *P. kesiya*, *P. yunnanensis* Franch.). In contrast, subsect. *Pinaster* Mayr ex Koehne is characterized by uni-nodal shoots and non-resinous buds. Leaves are borne in fascicles of two or three needles, with rare occurrences of mixed fascicles (as in *P. brutia* Ten.). Seed cones are larger, 6–20(–25) cm long, with thick, woody, and rigid seed scales, except in *P. heldreichii* Christ, where the scales are less rigid. The apophyses are typically lustrous brown, again with the exception of *P. heldreichii*. The umbo is unarmed or bears at most a minute, deciduous prickle, except in *P. pinaster* Aiton, where it is spinose (*e.g*., *P. latteri*, *P. merkusii*) (Farjon, 2010).

The taxonomic distinction between *Pinus latteri* and *P. merkusii* has long been a central issue in the taxonomy of Southeast Asian pines. Under the taxonomic concepts of Farjon (2005, 2010) and Businský (2008), *P. latteri* is widely distributed in mainland Southeast Asia, including Thailand, whereas *P. merkusii* is restricted to the Malesian region. Despite this, the name *P. merkusii* has historically been widely applied to Thai material without critical reassessment of voucher specimens.

Re-examination of Thai herbarium specimens previously identified as *P. merkusii* demonstrates that all examined material corresponds to *P. latteri*. These specimens consistently match the diagnostic morphological characters of *P. latteri*, and no examined material shows characters supporting identification as *P. merkusii*.

The results of this study therefore indicate that Thai populations previously reported as *P. merkusii* fall within the morphological variation of *P. latteri*. The misapplication of the name *P. merkusii* in Thailand appears to be historical and nomenclatural rather than reflecting the presence of two distinct taxa.

Based on the examined material in this study, all Thai specimens previously assigned to *P. merkusii* should be corrected to *P. latteri*. Our findings provide no evidence supporting the occurrence of *P. merkusii* among the Thai material investigated. Accordingly, the two-needle pine occurring in Thailand should be treated as *P. latteri* under the current taxonomic framework.

A comparative analysis of the morphological characters of *P. kesiya* and *P. latteri* in Thailand revealed slight differences from those reported in previous studies (Farjon, 2005, 2010; Li, 1997; Fu, Li & Mill, 1999; Phan et al., 2017; Averyanov et al., 2014). These minor variations may be attributable to differences in habitat and ecological conditions. Nevertheless, most characters were consistent with those described in earlier reports. Detailed comparisons are provided in Table 1.

According to Phengklai (1972), the seed cones of *P. kesiya* are rhomboid (or ovoid), whereas those of *P. latteri* (as *P. merkusii*) are described as ovoid or elongate-ovoid. However, our observations indicate considerable overlap in the shape and size of open seed cones between the two species. While the closed cones are similarly conical-ovoid, the length-to-width ratio provides a more reliable diagnostic character separating *P. kesiya* from *P. latteri*, with ratios of 1.1 to 1.9 and 2.0 to 2.9, respectively.

Previous studies have reported that *P. kesiya* has bi- or multi-nodal new foliage shoots, producing more than one whorl of branches or buds within a single growing season (Farjon, 2010; Li, 1997), whereas *P. latteri* has uni-nodal new foliage shoots (Farjon, 2010). However, new foliage shoots were not examined in the present study.

Farjon (2010) reported that the seed wings of *P. latteri* are not more than three times as long as the seed, whereas our observations indicate that they are 3 to 4.5 times as long as the seed.

The specimen *R. Geesink, T. Hattink & C. Phengklai 6560*, collected from a *Melaleuca* forest on a sandstone plateau at Khlong Kut, Trat Province, ca. 150 m a.m.s.l. (BKF [077328], L [L.1183259]), was identified by Aljos Farjon as *P. latteri* (annotated on the label as *P. merkusii*). However, the recorded habit and locality are inconsistent with the known ecology and distribution of *P. latteri*. This suggests that an error may have occurred in the specimen data recording.

Kim & Whang (1999) referred to *P. kesiya* under the name *P. khasya*, which is a synonym of *P. kesiya*, and to *P. merkusii* based on the specimen *T. Sørensen et al. 1729* (GH) cited from Vietnam and Thailand. Upon re-examination, we located this specimen at BKF; it was collected from Fang District, Chiang Mai Province, Thailand. Our investigation confirms that this specimen should be identified as *P. latteri*.

The anatomical characteristics of the needles of *P. kesiya* and *P. latteri* are largely similar, differing primarily in needle shape in transverse section. The needle of *P. kesiya* is triangular, whereas that of *P. latteri* is semicircular. These findings are consistent with previous reports (Farjon, 2005, 2010; Fu, Li & Mill, 1999; Averyanov et al., 2014).

According to Kim & Whang (1999), the stomatal shape of *P. kesiya* is elliptic, whereas that of *P. latteri* is rectangular. However, our observations indicate that the stomata of both species are elliptic. Kim & Whang (1999) also reported that the number of subsidiary cells in *P. kesiya* and *P. latteri* ranges from 6 to 8. In contrast, our examination shows that both species consistently have six subsidiary cells, comprising two polar subsidiary cells and four lateral subsidiary cells.

According to Kim & Whang (1999), the resin ducts of *P. kesiya* are positioned medially and externally, whereas those of *P. latteri* are internal and medial. However, our observations indicate that the resin ducts of *P. kesiya* are external, while those of *P. latteri* are medial.

## Conclusions

We have thoroughly examined specimens and present updated morphological descriptions and an identification key for two tropical pine species in Thailand, *Pinus kesiya* and *P. latteri*. Two names in *Pinus* are typified here: *P. kesiya* is designated with a neotype, and *P. kasya*, a synonym of *P. kesiya*, is lectotypified in a second-step.

*Pinus kesiya* and *P. latteri* can be clearly separated by bark characteristics, number of needles per fascicle, needle morphology, cone length-to-width ratio, apophysis shape, seed scale thickness, relative seed wing length, and the presence or absence of a grass stage. These traits provide reliable characters for identification in both field and herbarium studies.

In Thailand, *P. latteri* occurs at low to mid elevations (70–1,350 m a.m.s.l.) and is mainly associated with pine-deciduous dipterocarp forests and lower montane pine-oak forests, whereas *P. kesiya* occurs at higher elevations (800–1,800 m a.m.s.l.) and is mainly associated with lower montane pine-oak forests. Although both species occur at 800–1,350 m a.m.s.l., *P. latteri* occupies drier habitats, whereas *P. kesiya* is associated with more humid montane conditions.

The anatomical study revealed that *P. kesiya* and *P. latteri* share largely similar leaf structures, differing primarily in needle shape in transverse section. They also differ in the number and position of resin ducts.

Both species exhibit diagnostic features of the genus *Pinus*, including distinct rows of haplocheilic and sunken stomata, resin ducts, and plicate parenchyma. These structural characteristics reflect adaptations to xerophytic conditions and support their taxonomic placement within the genus.

The pollen morphology of *P. kesiya* and *P. latteri* in Thailand is highly similar. Both species exhibit monad, bisaccate, monosulcate pollen with oblate to oblate-spheroidal shapes and similar exine sculpturing (granulate *corpus* and perforate sacci). All measured characters show strong overlap between the two species, and *P. kesiya* shows slightly higher mean pollen size, with complete overlap in size ranges with *P. latteri*. Therefore, pollen morphology does not provide reliable diagnostic characters for distinguishing these species.

These palynological characteristics are diagnostic of conifers, particularly the presence of two air sacs (sacci) positioned on either side of the monosulcate aperture at the distal pole. This bisaccate condition represents a specialized structural adaptation to anemophily, increasing buoyancy and thereby enhancing dispersal efficiency.

This study provides a clarified and updated taxonomic framework for *Pinus* in Thailand, resolving nomenclatural issues and strengthening species delimitation through integrated morphological, anatomical, and palynological evidence. The recognition of reliable diagnostic characters facilitates accurate identification in both field and herbarium contexts and contributes essential baseline data for biodiversity assessments, forest management, and conservation planning. These findings also provide a foundation for future systematic, ecological, and evolutionary studies of tropical pines in Southeast Asia.

## Supplemental Information

10.7717/peerj.21679/supp-1 Supplemental Information 1 Figures & Additional specimen examined.

## Additional Information and Declarations

### Field Study Permissions

The following information was supplied relating to field study approvals (*i.e*., approving body and any reference numbers):

Permission to collect specimens of *Pinus* was granted by the Department of National Parks, Wildlife and Plant Conservation (Permit No. MNRE 0907.404/25551; 7 December 2023) and the Forest Industry Organization (FIO) (Permit No. MNRE 1408/285; 15 January 2026).

### Data Availability

The following information was supplied regarding data availability:

The data is available in the manuscript under the Materials & Methods and Results sections.

The data is available in the Supplemental File.
