# Supplementary material for: A taxonomic revision of Pinus (Pinaceae) in Thailand
Source: PeerJ. 2026 Sep 3;14:e21679. doi: 10.7717/peerj.21679 (PMC13549149; doi:10.7717/peerj.21679)
Supplement: Supplementary file 1 [file peerj-14-21679-s001.docx]

**Supplementary 1**

**Figure 1** **Measurements of bisaccate pollen of the genus *Pinus*.** (PL = pollen length with sacci; PW = pollen width with sacci; CL = corpus length; CW = corpus width; SL = saccus length; SW = saccus width; DS = distance between sacci). Photo: Chatchai Ngernsaengsaruay and Pichet Chanton.

**Figure 2** ***Pinus kesiya*.** (A) Stem and outer bark. (B and F) Branchlets, leaves, and pollen cones. (C) Branchlets, leaves, and open seed cones. (D) Branchlet showing scars of fallen needle fascicles. (E) Needle fascicle. (G) Closed seed cone. (H) Open seed cone. (I) Seed scales. (J) Seed scale and winged seeds. (K) Winged seed. (L) Seedling with several cotyledons. Photo: drawn by Wanwisa Bhuchaisri.

**Figure 3** ***Pinus kesiya*.** (A and B) Habit. (C) Stem and outer bark. (D–F) Branchlets, leaves, and pollen cones. (G) Branchlets, leaves, pollen cones, and ovulate cone. (H) Branchlets, leaves, and ovulate cones. (I–J) Branchlets, leaves, and seed cones (closed and open). (K) Open seed cones. (L) Winged seeds. (M) Seedlings with several cotyledons. (N) Seedling without a grass stage. Photos: Chatchai Ngernsaengsaruay (A–K and M–N); Pichet Chanton (L).

**Figure 4** **Distribution of two Thai *Pinus* species.** (A) *Pinus kesiya* is distributed in India (Assam), Myanmar, China (Yunnan; Xizang), Vietnam, Laos, Cambodia, Thailand, and the Philippines (Luzon). In Thailand, this species is distributed in the northern, north-eastern, eastern, and south-western regions. (B) *Pinus latteri* is distributed in Myanmar, China (Guangdong; Guangxi), Vietnam, Laos, Cambodia, and Thailand. In Thailand, this species is distributed in the northern, north-eastern, eastern, south-western, and central regions. Maps: Pichet Chanton and Chatchai Ngernsaengsaruay.

**Figure 5** **Habitats and ecology of *Pinus* *kesiya* in Thailand.** (A) Lower montane pine-oak forest at Doi Langka, Chiang Rai Province, at an elevation of ca. 1,700 m. a.m.s.l. (B) Lower montane pine-oak forest at Phu Luang, Loei Province, at an elevation of ca. 1,490 m. a.m.s.l. (C) Lower montane pine-oak forest at Doi Chiang Dao, Chiang Mai Province, at an elevation of ca. 1,375 m. a.m.s.l. (D–F) Lower montane pine-oak forest (pine-oak savanna-like structure) at Phu Soi Dao, Uttaradit Province, at an elevation of ca. 1,600 m. a.m.s.l. Photos: Chatchai Ngernsaengsaruay.

**Figure 6** ***Pinus latteri*.** (A) Stem and outer bark. (B) Branchlets, leaves, pollen cones, and closed seed cone. (C) Branchlet and leaves. (D) Branchlet showing scars of fallen needle fascicles. (E) Needle fascicle. (F)Pollen cones. (G) Closed seed cone. (H) Open seed cone. (I) Seed scales. (J) Seed scale and winged seeds. (K) Winged seed. Photo: drawn by Wanwisa Bhuchaisri.

**Figure 7** ***Pinus latteri*.** (A) Habit. (B–E) Stems and outer bark. (F) Branchlets, leaves, and pollen cones. (G) Outer bark, branchlets, leaves, pollen cones, and closed seed cones. (H) Branchlets, leaves, and closed seed cones. (I–J) Open seed cones. (K) Winged seeds (L) Seedling with several cotyledons. (M) Seedling with a grass stage. Photos: Chatchai Ngernsaengsaruay (A–J and L–M) and Pichet Chanton (K).

**Figure 8** **Habitats and ecology of *Pinus* *latteri* in Thailand.** (A) Lower montane pine-oak forest at Phu Kradueng, Loei Province, at an elevation of ca. 1,200 m. a.m.s.l. (B) Lower montane pine-oak forest at Phu Hin Rong Kla, Phitsanulok Province, at an elevation of ca. 1,100 m. a.m.s.l. (C–D) Lower montane pine–oak forest (pine–oak savanna-like structure) at Phu Kradueng, Loei Province, at an elevation of ca. 1,200 m a.m.s.l. (E–F) Lower montane pine-oak forest (pine-oak savanna-like structure) at Phu Luang, Loei Province, at an elevation of ca. 1,250 m. a.m.s.l. (G) Pine-deciduous dipterocarp forest at Thung Salaeng Luang, Phetchabun Province, at an elevation of ca. 700 m a.m.s.l. (H) Pine-deciduous dipterocarp forest at Thung Non Son, Phitsanulok Province, at an elevation of ca. 950 m a.m.s.l. (I) Pine-deciduous dipterocarp forest at Mae Chaem, Chiang Mai Province, at an elevation of ca. 770 m a.m.s.l. (J) Pine-deciduous dipterocarp forest at Phu Ruea, Loei Province, at an elevation of ca. 1,200 m a.m.s.l. (K) Pine-deciduous dipterocarp forest at Phu Toei, Suphan Buri Province, at an elevation of ca. 750 m a.m.s.l. (L) Pine-deciduous dipterocarp forest at Pha Chana Dai, at an elevation of ca. 350 m a.m.s.l. Photos: Chatchai Ngernsaengsaruay.

**Figure 9 Leaf anatomy of *Pinus kesiya*.** (A, F−H) Transverse section. (B) Adaxial epidermis (under SEM). (C) Abaxial epidermis (under SEM). (D−E) Abaxial epidermis (under LM). (Abe = abaxial epidermis, Ade = adaxial epidermis, Ct = cuticle, EpiC = epithelial cell, Ed = endodermis, EpC = epidermal cells, Hp = hypodermis, LSubC = lateral subsidiary cells, Me = Mesophyll, P = phloem, Pa = parenchyma, PliPa = plicate parenchyma, PSubC = polar subsidiary cells, Rd = resin duct, ShC = sheath cell, St = stoma, Tt = transfusion tissue, Vb = vascular bundle and X = xylem). Photos: Pichet Chanton and Chatchai Ngernsaengsaruay.

**Figure 10 Leaf anatomy of *Pinus latteri*.** (A, F−H) Transverse section. (B) Adaxial epidermis (under SEM). (C) Abaxial epidermis (under SEM). (D−E) Abaxial epidermis (under LM). (Abe = abaxial epidermis, Ade = adaxial epidermis, Ct = cuticle, EpiC = epithelial cell, Ed = endodermis, EpC = epidermal cells, Hp = hypodermis, LSubC = lateral subsidiary cells, Me = Mesophyll, P = phloem, Pa = parenchyma, PliPa = plicate parenchyma, PSubC = polar subsidiary cells, Rd = resin duct, ShC = sheath cell, St = stoma, Tt = transfusion tissue, Vb = vascular bundle and X = xylem). Photos: Pichet Chanton and Chatchai Ngernsaengsaruay.

**Figure 11 LM and SEM micrographs of pollen grains of *Pinus kesiya*.** (A and D) Bisaccate pollen in equatorial view. (B and E) Corpus in polar view. (C and F) Sacci and aperture in polar view. (G) Exine sculpturing on the corpus. (H) Exine sculpturing on the sacci. (A–C under LM; D–H under SEM). Photos: Pichet Chanton (under LM); Chatchai Ngernsaengsaruay and Pichet Chanton (under SEM).

**Figure 12 LM and SEM micrographs of pollen grains of *Pinus latteri*.** (A and D) Bisaccate pollen in equatorial view. (B and E) Corpus in polar view. (C and F) Sacci and aperture in polar view. (G) Exine sculpturing on the corpus. (H) Exine sculpturing on the sacci. (A–C under LM; D–H under SEM). Photos: Pichet Chanton (under LM); Chatchai Ngernsaengsaruay and Pichet Chanton (under SEM).

‘

**Supplementary 2**

***Pinus kesiya***

**Additional specimens examined. THAILAND. Northern: Mae Hong Son** [Pang Ma Pha, seed cone, 2 May 2009, *M. Hilscher 38* (L [L.4320773]); Huai Nam Dang National Park, ♂ cone, 5 Feb 2013, *M. Norsaengsri 10144* (BKF); Huai Wai Forest Ranger Unit (Temporary), Mae Lao-Mae Sae Wildlife Sanctuary, Pai District, lower montane pine-oak forest, ca. 1,460 m a.m.s.l., 7 Nov 2022, *C. Ngernsaengsaruay* personal observation with photos; Pai District, lower montane pine-oak forest, ca. 1,160 m a.m.s.l., 27 May 2023, *C. Ngernsaengsaruay* personal observation with photos]; **Chiang Mai** [Doi Suthep, seed cone, 1 Jan 1905, *C. C. Hosseus 318* (K [K000554160], L [L.1183133], M [M-0168819, M-0168820]); ibid., seed cone, 30 Jan 1910, *A. F. G. Kerr 962* (K [K000554162]); ibid., seed cone, 1 Nov 1920, *J. F. Rock 163* (US [02065554]), *J. F. Rock 247* (US [02065566]); ibid., ♂ cone, 28 Sep 1949, *Ploenchit 48* (BKF); ibid., seedling, 4 May 1958, *T. Sørensen et al. 3235* (BKF); ibid., seed cone, 29 Dec 1988, *J. F. Maxwell 88-1416* (BKF, L [L.4178500, L.4178501]); ibid., ♂ cone, 2 Feb 1989, *J. F. Maxwell 89-141* (BKF, L [L.1183161], MO [976290], UF [268802]); Khun Chang Khian, seed cone, 13Mar 1930, *Phraya Phananuchon 4* (BKF); Doi Pui, seed cone, 27 Nov 1984, *H. Koyama T-49525* (BKF); ibid., seed cone, May 1987, *C. Phengklai et al. 6330* (BKF); ibid., 8 Aug 1988, *M. N. Tamura T-60755* (BKF); Doi Suthep-Pui National Park, seed cone, 14 Jul 1988, *H. Koyama T-61910* (BKF); ibid., ♂ cone & seed cone, 10 Feb 1999, *C. Kuarak 13* (L [L.4169890]); Doi Pui, lower montane pine-oak forest, ca. 1,400 m a.m.s.l., 24 Apr 2012, *C. Ngernsaengsaruay* personal observation with photos; Doi Inthanon National Park, 16 Jan 1905, *C. C. Hosseus 347b* (M [M-0168821]); ibid., 28 Sep 1949, *P. Suvarnakoses 48* (BR [BR0000026050065V]); Mae Chaem, 25 Jan 1964, *B. Hansen et al. 11110* (L [L.1183118]); Mae Pan Waterfall, seed cone, 29 Jul 1988, *N. Fukuoka T-62312* (BKF); Chom Thong District, ♂ cone, 15 Jan 1993, *J. F. Maxwell 93-40* (BKF, L [L.4178640, L.4178641]); ibid., ♂ cone, 3 Feb 1998, *F. Konta & C. Phengklai 3911* (BKF); ibid., 6 May 2010, *P. Georgiadis 315* (L [L.2058679]); Doi Inthanon, ♂ cone, 17 Dec 1993, *C. Yonebayashi 93039* (BKF); ibid., ♂ cone, 3 Feb 1998, *F. Konta & S. Khao-iem* 10822 (BKF); Doi Pha Tang, Doi Inthanon National Park, lower montane pine-oak forest, ca. 1,040 m a.m.s.l., seed cone, 25 May 2023, *C. Ngernsaengsaruay* personal observation with photos; Doi Inthanon National Park, lower montane pine-oak forest, ca. 1,500 m a.m.s.l., 25 May 2023, *C. Ngernsaengsaruay* personal observation with photos; Doi Chiang Dao, ♂ cone, 24 Dec 1931, *Put 4529* (K [K000554199]); ibid., 30 Jul 1968, *K. Larsen et al.* *2852* (BKF); ibid., seed cone, 15 Nov 1988, *W. Nanakorn & H. T. Beck 880171* (MO [103464246], NY [00039940]); ibid., 23 Oct 1992, *R. Pooma 696* (BKF); ibid., 1 Mar 1995, *J. F. Maxwell 95-165* (BKF, L [L.4178639]); San Pa Kia, 23 Jan 1981, *Forestry student s.n.* (BKF [72298]); Doi Chiang Dao, lower montane pine-oak forest, ca. 1,375 m a.m.s.l., 3 Dec 2002, *C. Ngernsaengsaruay* personal observation with photos; Doi Ang Khang, seed cone, 26 May 1998, *T. Wongprasert s.n.* (BKF [123797]); Ban Huai Khiat Haeng, Kalayaniwattana District, 8 Mar 2018, *W. Pongamornkul 6594* (BKF)]; **Chiang Rai** [Doi Tung, ♂ cone, 14 Feb 1983, *H. Koyama et al. T-33532* (BKF); Khun Chae National Park, seed cone, 12 Nov 1997, *U. Wongchai WU039* (BKF); Khun Chae National Park, Wiang Pa Pao District, pine-deciduous dipterocarp forest, 920–930 m a.m.s.l., seed cone, 28 May 2023, *C. Ngernsaengsaruay* personal observation with photos; Doi Langka nature trail (Doi Pha Ngom, Doi Langka Noi), Khun Chae National Park, Wiang Pa Pao District, lower montane pine-oak forest, 1,540–1,770 m a.m.s.l., 26–28 Dec 2025, *C. Ngernsaengsaruay* personal observation with photos]; **Phayao** [Tham Prakai Phet, Mae Chai District, seed cone, 26 Jul 2016, *N. Muangyen 254* (BKF); Doi Luang National Park, ♂ cone, 9 Feb 2016, *N. Muangyen 663* (BKF)]; **Lamphun** [Doi Khun Tan National Park, Mae Tha District, seed cone, 5 Sep 1967, *M. Tagawa et al. T-9260* (L [L.1183124]); ibid., ♂ cone, 31 Jan 1994, *J. F. Maxwell 94-158* (BKF, L [L.4178589]); Doi Chang, Ban Pa Pae, Pa Phlu Subdistrict, Ban Hong District, pine-deciduous dipterocarp forest, ca. 1,100 m a.m.s.l., 22 Apr 2012, *C. Ngernsaengsaruay* personal observation with photos]; **Lampang** [Chae Son National Park, ♂ cone & seed cone, 31 Jan 1997, *F. Maxwell 97-97* (BKF, L [L.4169572], MO [1775345]); Doi Luang National Park, Wang Nuea District, seed cone, 10 Dec 2002, *I. Schanzer N02-115* (BKF)]; **Uttaradit** [Phu Soi Dao National Park, seed cone, 30 Nov 20xx, *N. Pan-in PSD013* (BKF); ibid., ♂ cone, 22 Feb 2018, *N. Pan-in PSD026* (BKF); Phu Soi Dao National Park, Nam Pat District, lower montane pine-oak forest, ca. 1,600 m a.m.s.l., 4 Jan 2025, *C. Ngernsaengsaruay personal observation with photos*]; **Phitsanulok** [Air Raid Shelter, Phu Hin Rong Kla National Park, Nakhon Thai District, lower montane pine-oak forest, ca. 1,200 m a.m.s.l., ♂ cone & seed cone 2 Feb 2007, *C. Ngernsaengsaruay* personal observation with photos]; **North-Eastern:** **Phetchabun** [Nam Nao, ♀ cone, 1 Apr 1953, *Sanong 36* (BKF); Locality unspecified, ♀ cone, 10 Apr 1976, *B. Sangkhachand & C. Phengklai 3118*, (BKF, L [L.1183134], K [K000554158]); Nong Mae Na–Thung Nang Phaya route, Thung Salaeng Luang National Park, Khao Ko District, pine-deciduous dipterocarp forest, 750–900 m a.m.s.l., 29 Jan 2007, *C. Ngernsaengsaruay* personal observation with photos]; **Loei** [Phu Kradueng National Park, ♀ cone, 12 Mar 1924, *A. F. G. Kerr 8740* (K [K000554201]); ibid., 12 Jan 1960, *L. B. Abbe et al. 9464* (BKF, NY [4278102]); ibid., seed cone, 28 Nov 1965, *M. Tagawa et al. T-507* (BKF, L [L.1183125]); ibid., seed cone, 30 Nov 1965, *M. Tagawa et al. T-959* (BKF, L [L.1183126]); ibid., 19 Dec 1982, *H. Koyama et al. T-31369* (BKF); ibid., seed cone, 2 Nov 1984, *G. Murata et al. T-42876* (BKF); ibid., seed cone, 4 Sep 1988, *H. Koyama T-61543* (L [L.4168389]); ibid., seed cone, 26 Aug 1954, *T. Smitinand 1881* (BKF); Phu Kradueng National Park, lower montane pine-oak forest, ca. 1,200 m a.m.s.l., seed cone, 13 Nov 2021, *C. Ngernsaengsaruay* personal observation with photos; Phu Ruea National Park, lower montane pine-oak forest, ca. 1,360 m a.m.s.l., 12 Jun 2023, *C. Ngernsaengsaruay* personal observation with photos; Phu Luang, ♂ cone & seed cone, 1 Oct 1966, *E. Hennipman 3601* (K [K000554159], L [L.1183123]); ibid., seed cone, 8 Jun 2015, *S. Tagane T4491* (BKF); Phu Luang Wildlife Sanctuary, lower montane pine-oak forest, 1,470–1,490 m a.m.s.l., seed cone, 10 Jun 2023, *C. Ngernsaengsaruay* personal observation with photos].

***Pinus latteri***

**Additional specimens examined. THAILAND. Northern:** **Mae Hong Son** [Locality unspecified, s.d., *Unknown s.n.* (CS [195370]); Tham Pla–Namtok Pha Suea National Park, seed cone, 11 Nov 2013, *C. Lakoet 0442* (BKF)]; **Chiang Mai** [Chiang Dao, 3 Mar 1957, *Khanchai 393* (BKF); Fang District, 27 Feb 1958, *T. Sørensen et al. 1729* (BKF); Bo Luang, seed cone, 13 Mar 1967, *E. N. Cooling 324* (K [K000554172]); ibid., ♂ cone, 13 Mar 1967, *E. N. Cooling 325* (K [K000554168]), *E. N. Cooling 326* (K [K005457247]); ibid., seedling with a grass stage, 14 Mar 1967, *E. N. Cooling 330* (K [K000554171]); Doi Suthep, ♂ cone, 21 Mar 1951, *T. Smitinand 159* (US [2036221]); ibid., 8 Feb 1957, *T. Smitinand 3774* (BKF); Doi Inthanon National Park, Mae Chaem District, ♂ cone & seed cone, 11 Feb 1998, *F. Konta et al. 4232* (BKF); Doi Inthanon, 2 Dec 1998, *M. Hara et al. 927* (BKF); ibid., ♂ cone & seed cone, 11 Feb 1998, *F. Konta & S. Khao-iem 10995* (BKF); Doi Inthanon National Park, Ban San Phatthana, Mae Chaem District, pine-deciduous dipterocarp forest, ca. 770 m a.m.s.l., seed cone, 24 May 2023, *C. Ngernsaengsaruay* personal observation with photos; Doi Inthanon National Park, Ban Mae Pa Ko, Chom Thong District, pine-deciduous dipterocarp forest, ca. 630 m a.m.s.l., 25 May 2023, *C. Ngernsaengsaruay* personal observation with photos; Mae Taeng District, lower montane pine-oak forest, 1,030–1,080 m a.m.s.l., 26 May 2023, *C. Ngernsaengsaruay* personal observation with photos]; **Chiang Rai** [Huai San, ♂ cone, 7 Mar 1912, *A. F. G. Kerr 2506* (K [K000554165]); between Mae Suai and Huai San, 5 Jan 1922, *J. F. Rock 1936* (US [1330139]); Wiang Pa Pao District, pine-deciduous dipterocarp forest, ca. 830 m a.m.s.l., 1 Feb 2013, *C. Ngernsaengsaruay* personal observation with photos; Khun Chae National Park, Wiang Pa Pao District, pine-deciduous dipterocarp forest, 920–930 m a.m.s.l., seed cone, 28 May 2023, *C. Ngernsaengsaruay* personal observation with photos]; **Phayao** [Ban Wang Tham, Chiang Kham District, 25 Jun 1954, *T. Smitinand 1649* (BKF)]; **Lamphun** [Doi Khun Tan National Park, Mae Tha District, ♂ cone, 28 Feb 1994, *J. F. Maxwell 94-278* (BKF, L [L.4178594]); Doi Chang, Ban Pa Pae, Pa Phlu Subdistrict, Ban Hong District, pine-deciduous dipterocarp forest, ca. 1,100 m a.m.s.l., seed cone, 22 Apr 2012, *C. Ngernsaengsaruay* personal observation with photos]; **Lampang** (Doi Chong National Park, ♀ cone & seed cone, Jun 1933, *I. Churuethai 2* (BKF)]; **Tak** [Mae Sot, seed cone, 15 Mar 1967, *E. N. Cooling 334* (K [K000554166]); Huai Sak Ngam Forest, Li District, s.d., *Saisaeng 7* (BKF)]; **Phitsanulok** (Lan Hin Taek, Phu Hin Rong Kla National Park, Nakhon Thai District, lower montane pine-oak forest, ca. 1,100 m a.m.s.l., ♀ cone & seed cone, 25 Apr 2007, *C. Ngernsaengsaruay* personal observation with photos; Thung Non Son, Thung Salaeng Luang National Park, Chom Phu Subdistrict, Noen Ma Prang District, pine-deciduous dipterocarp forest, ca. 950 m a.m.s.l., seed cone, 16 Oct 2007, *C. Ngernsaengsaruay* personal observation with photos]; **North-Eastern:** **Phetchabun** [Thung Salaeng Luang National Park, seed cone, 25 Jul 1973, *G. Murata et al. T-17134* (BKF, L [L.1183262]); Nong Mae Na–Thung Nang Phaya route, Thung Salaeng Luang National Park, Khao Ko District, pine-deciduous dipterocarp forest, 700–900 m a.m.s.l., seed cone, 29 Jan 2007, *C. Ngernsaengsaruay* personal observation with photos; Locality unspecified, 4 Jan 1960, *T. Smitinand 6288* (BKF)]; **Loei** [Phu Kradueng National Park, 12 Mar 1924, *A. F. G. Kerr 8739* (K [K000554200]); ibid., 21 Sep 1934, *T. Smitinand 1976* (BKF); ibid., 23 Jul 1948, *Din 61* (BKF); ibid., 29 Mar 1949, *D. Bunpheng 290* (BKF, BR [BR0000026050072V]); ibid., ♂ cone, 11 Feb 1952, *D. Bunpheng 479* (BKF); ibid., 9 Jul 1959, *T. Smitinand 5879* (BKF); ibid., 12 Jan 1960, *E. C. Abbe et al. 9465* (BKF, K [K000554164], NY [4278112]); ibid., 11 Sep 1963, *T. Smitinand & H. Sleumer 4780* (L [L.1183260]); ibid., 7 Nov 1970, *C. Charoenphol et al. 4696* (BKF); ibid., 11 Mar 1973, *T. Smitinand 11800* (BKF); ibid., 2 Nov 1984, *G. Murata et al. T-42788* (BKF); ibid., seed cone, 22 Jun 2015, *S. Tagane et al. T4519* (BKF); ibid., seedling with a grass stage, 8 Jun 2020, *T. Thananthaisong et al. 14* (BKF); Phu Kradueng National Park, lower montane pine-oak forest, ca. 1,200 m a.m.s.l., seed cone, 27 Oct 2013 and 13 Nov 2021, *C. Ngernsaengsaruay* personal observation with photos; Phu Ruea National Park, 19 Jan 2010, *M. S. Nuraliev & I. A. Savinov 421* (MW [MW0732137]); Phu Ruea National Park, pine-deciduous dipterocarp forest, 1,170–1,200 m a.m.s.l., seed cone, 12 Jun 2023, *C. Ngernsaengsaruay* personal observation with photos; Khok Huai Toei, Phu Luang Wildlife Sanctuary, lower montane pine-oak forest, 1,230–1,250 m a.m.s.l., seed cone, 11 Jun 2023, *C. Ngernsaengsaruay* personal observation with photos]; **Eastern:** **Surin** [Sangkha District, 12 Jan 1924, *A. F. G. Kerr 8263* (K [K000554202])]; **Si Sa Ket** [Nong I Kwang, Kanthararom District, 13 Jul 1951, *Unreadable 123* (BKF[10759]); Ban Nong Pha Naeng, Nam Kliang District, ♂ cone & seed cone, 11 Mar 1967, *S. Phusomsaeng 50* (BKF, K [K000554203], L [L.1183257])]; **Ubon Ratchathani** [Ban Ba Hai, Khong Chiam District, seed cone, 20 Aug 1986, *C. Niyomdham 1238* (BKF); Pha Cha Na Di, Dong Na Tham Forest, Pha Taem National Park, Na Pho Klang Subdistrict, Khong Chiam District, pine-deciduous dipterocarp forest, ca. 350 m a.m.s.l., 12 Nov 2005, C*. Ngernsaengsaruay* personal observation with photos]; **South-Western:** **Phetchaburi** [Tha Yang District, seed cone, 16 Feb 2006, *D. J. Middleton et al. 3709* (BKF, E [E00261729], K [K000577002], L [L.4178643]); Khao Son Community Forest, Khao Ka Puk Subdistrict, Tha Yang District, pine-deciduous dipterocarp forest, ca. 70 m a.m.s.l., 7 Jul 2023, *C. Ngernsaengsaruay* personal observation with photos]; **Central: Suphan Buri** [Phu Toei National Park, seed cone, 29 Jun 2001, *T. Wongprasert 016-14* (BKF); Phu Toei National Park, Dan Chang District, pine-deciduous dipterocarp forest, ca. 750 m a.m.s.l., seed cone, 28 Jul 2023, *C. Ngernsaengsaruay* personal observation with photos].

A comparison of the morphological characteristics of *P. kesiya* and *P. latteri* in Thailand with those reported in previous studies is presented in Table 1.
